# Flexible navigation with neuromodulated cognitive maps

**DOI:** 10.1371/journal.pcbi.1013487

**Published:** 2026-07-28

**Authors:** Krubeal Danieli, Mikkel Elle Lepperød

**Affiliations:** 1 Center for Integrative Neuroplasticity, FYSCELL, University of Oslo, Oslo, Norway; 2 Simula Research Laboratory, Oslo, Norway; Ghent University, BELGIUM

## Abstract

Animals develop specialized cognitive maps during navigation, constructing environmental representations that facilitate efficient exploration and goal-directed planning. The hippocampal CA1 region is implicated as the primary neural substrate for cognitive mapping, housing spatially tuned cells that adapt based on behavioral patterns and internal states. Computational approaches to modeling these biological systems have employed various methodologies. Although labeled graphs with local spatial information and deep neural networks have provided computational frameworks for spatial navigation, significant limitations persist in modeling one-shot adaptive mapping. We introduce a biologically inspired place cell architecture that develops cognitive maps during exploration of novel environments. Our model implements a simulated agent for reward-driven navigation that forms spatial representations online. The architecture incorporates behaviorally relevant information through neuromodulatory signals that respond to environmental boundaries and reward locations. Learning combines rapid Hebbian plasticity, lateral competition, and targeted modulation of place cells. Analysis of the model across a variety of environments demonstrates that online map formation and reward-directed navigation can emerge within a single simulated trial, without the multi-epoch training typically required by reinforcement-learning approaches. The simulation results show that the agent successfully explores and navigates to target locations in various environments, adapting when reward positions change. Analysis of neuromodulated place cells reveals dynamic changes in neuronal density and place field size after behaviorally significant events. These findings align with experimental observations of reward effects on hippocampal spatial cells while providing computational support for the efficacy of biologically inspired approaches to cognitive mapping.

## Author summary

This work proposes a biologically inspired architecture for neuromodulated cognitive maps that addresses key limitations in current approaches, such as offline training, biologically unrealistic learning, and reliance on external coordinate inputs. The model constructs spatial representations through place cells generated dynamically during navigation, with learning mediated by neuromodulatory signals that integrate boundaries and reward-related information. Simulations show that the model supports navigation, flexible path planning, and robustness to environmental changes. The analysis reveals adaptive strategies including context-dependent regulation of place cell density and place field scaling, aligned with experimental findings from hippocampal CA1. This proof-of-concept highlights how plausible neural dynamics and computationally advantageous strategies can emerge from simple modeling assumptions.

## 1. Introduction

Survival in complex environments requires efficient navigational strategies. From desert ants to humans, successful wayfinding, defined as navigating toward goals that are not directly visible, depends on emergent internal spatial representations, known as cognitive maps [1,2]. Understanding how such maps are constructed from ongoing experiences and how they can be exploited for flexible goal-directed navigation remains an active area of research in both neuroscience and reinforcement learning.

In the brain, the hippocampus (HP) and the entorhinal cortex (EC) are the central areas involved in spatial representation. They contain specialized neurons encoding spatial and contextual information, including grid, border, speed, and place cells [3–5]. In particular, the latter are numerous in the CA1 hippocampal sub-region and have attracted interest due to the convergence of inputs from entorhinal grid cells, the CA3 hippocampal sub-region, and the lateral EC [6–9]. This strategic integration of diverse spatial and contextual signals suggests that CA1 place cells may play a critical role in the formation and maintenance of cognitive maps [10].

Traditional theories of cognitive maps suggest that spatial representations can emerge from multiple navigational strategies. Early frameworks suggested that the hippocampus encodes both spatial location and direction [11], while graph-based models captured structural aspects of spatial organization, although often at the cost of scalability [12].

Another approach is path integration, which involves tracking one’s position by integrating past movements—using actions or velocity vectors—to estimate the trajectory and return to the point of origin.

An important strength of path integration lies in its independence from external cues, relying instead on idiothetic internal-motion signals, which are considered biologically plausible and believed to involve entorhinal grid cells [13,14]. A common computational methodology to investigate path integration involves recurrent neural networks combined with linear readouts. When trained in navigation tasks, these systems spontaneously develop spatially tuned activity patterns reminiscent of grid, place, and border cells [15–17].

Another proposal is the Tolman-Eichenbaum machine (TEM), a model that generalizes across spatial and relational tasks while reproducing key biological neural activity patterns [18–20]. Learning in TEM occurs through gradient descent over many episodes or epochs, in contrast to adaptive, context-dependent learning observed in biological systems. As with several modeling approaches, its neuronal dynamics remain simplified, typically reduced to artificial neurons defined by weighted sums and fixed non-linear activation functions. An alternative is the successor representation framework (SR), built around the learning of a predictive map of the explored state space, which can be used for active navigation [21,22]. It has been linked to hippocampal cognitive map generation, with the possibility of incorporating reward information and formulation in terms of biologically plausible mechanisms [23–26].

Neuromodulation is another relevant element in brain dynamics, affecting physiology, cognition, and behavior [27]. Its functions include filtering meaningful internal and external signals, influencing neuronal dynamics, and learning by altering synaptic states and parameters [28–30]. An important neuromodulator is dopamine, long associated with reward information, prediction error encoding [30,31], and novelty detection [29], mechanisms closely related to reinforcement learning principles [32,33]. In addition, projections from the Ventral Tegmental Area (VTA) and Locus Coeruleus (LC) have been shown to target the hippocampal circuits [34–36], to be involved in memory formation [37], and affect the adjustment of CA1 place cells [38–41], particularly through LEC inputs [42,43].

Other approaches have incorporated neuromodulators with reward-driven Hebbian plasticity [44] or other learning features, such as sparsity and update scaling [45]. However, these architectures often remain limited in their ability to integrate these components into a biologically grounded framework that works without extensive training, gradient-based optimization, or external coordinate inputs while still providing adaptable planning and behavior.

In this work, we addressed these limitations by proposing a bio-inspired model of cognitive map formation learned online from sensory experiences. The model builds on the emergence of place cell representations from grid cells activity and velocity information, that is, internally available idiothetic input, in line with the path integration approach [15,16]. We designed plasticity mechanisms through which neuromodulatory signals could target and modulate place cells, augmenting the resulting map with behaviorally relevant information. Combined with a network-based path planning mechanism, our architecture enabled an artificial agent to construct content-rich topological maps of its environment on-the-fly and to leverage them for targeted navigation. We took direct inspiration from previous work discussing the involvement of neuromodulation in hippocampal spatial processing, for example, in terms of the gating of incoming input streams and the influence of synaptic plasticity [46–48].

Our primary aim was to evaluate the potential of the emergent cognitive map to support navigation in unexplored environments, including the ability to record the location of a reward and to generate paths to it from already visited regions. Notably, the model begins without any spatial or qualitative knowledge of its surroundings; such knowledge is acquired incrementally as the agent moves. The lack of separation between map construction and reward collection ensures that the task operates entirely online.

A second aim was to assess the model’s robustness and adaptability to qualitative and spatial changes, such as shifts in reward location or the introduction of new boundaries. Additionally, the contribution of neuromodulation to the regulation of place cell activity was also examined as experience accumulated, investigating the effect on cell density and the size of place fields. Finally, we sought to quantify the effect of neuromodulation on task performance through a series of comparative ablation experiments.

These analyses allowed us to characterize the effects of neuromodulation on place field properties, such as modulation of their size and density, and to discuss potential links to experimental findings [49,50].

The remainder of the paper is organized as follows: Section 2 details the model and experimental setup; Section 3 presents results; Section 4 discusses the broader implications and future directions.

## 2. Methods

We propose a model of cognitive map formation driven by an agent’s experience within a closed environment.

**Fig 1:**
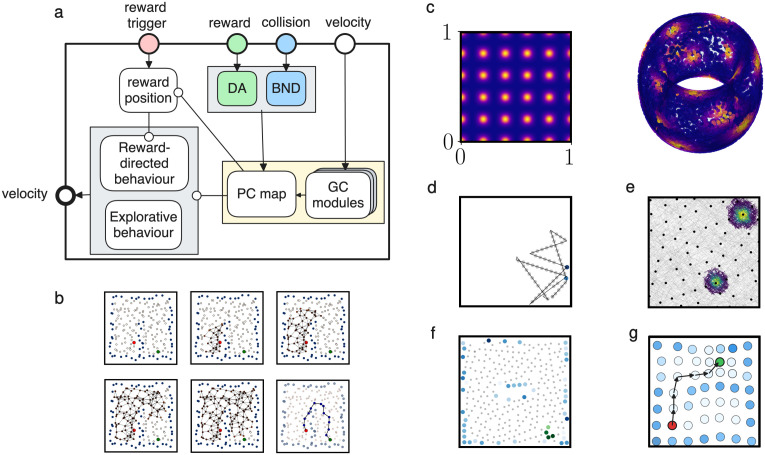
Model layout and spatial representations. **a**: layout of the architecture consisting of four main inputs: a reward signal targeting the dopamine modulation component (DA), a collision signal targeting the boundary modulation component (BND), a velocity vector (the actual movement made by the agent) aiming at the grid cells module, and a reward trigger acting as a flag to enable reward-directed navigation. A place cells component represents where a cognitive map actually resides, and receives input from the grid cells for spatial information, modulators for non-spatial data, and it is used for navigation and determining the reward position. Two policy components are responsible for determining the agent’s action: an explorative behaviour and a reward-directed behaviour, the latter being conditioned on the ability to calculate the reward position and the presence of a trigger signal. **b**: visualization of part of the path-finding algorithm, propagation of an activity wave through the place cell network from top-left to bottom-center, and the calculated path visualization in the bottom-right. **c**: a module of grid cells defined in a bounded square space of length 1, and an activity representation of their receptive field over a torus. **d**: formation of a cognitive map during navigation, with place cells (grey) being formed over the agent’s trajectory (black line); boundary-modulated place cells are formed near boundaries (blue). **e**: spatial distribution of the place cell centers, together with the place fields of two cells. **f**: representation of the cognitive map as place cells tagged by neuromodulators during collisions (blue), reward events (green), or untagged (grey); the color gradient correlates with the intensity of the neuromodulators’ weights. **g**: illustration of a path (arrows) over a place cell map connecting an initial position (red) and a goal (green).

The architecture operates with minimal external inputs, limited to binary reward and collision signals, as illustrated in Fig 1A. Instead of relying on exteroceptive cues, spatial representations emerge from idiothetic information, that is, the agent’s internal perception of self-motion [51], consistent with path-integration frameworks. In practice, we use the agent’s ground truth velocity vector, that is, its actual displacement within the environment, as the primary navigational signal, reflecting the integration of inertial and proprioceptive signals observed in biological systems [13,52]. Since no visual information is used, the agent effectively navigates in the dark. Importantly, the model does not receive allocentric coordinates as sensory input; any two-dimensional positions used for planning or visualization are derived from the internally constructed place cell representation.

### 2.1. Place cell formation

The primary spatial representation is formed by a set of simplified grid cell modules, each encoding a periodic tiling of 2D space, which directly maps to a toroidal manifold ${𝐓}^{2}$ (Fig 1C). Departing from traditional grid cell modeling approaches [53,54], we generate population activity directly by Gaussian tuning on a torus, continuously updated using the agent velocity vector, an approach used in previous work [8].

The grid cell population vector ${𝐮}^{\text{GC}}$ is forwarded to a place cell network with initial zero synaptic weights. When no place cell is sufficiently active for a given input, a silent unit is randomly selected and imprinted with the current grid activity pattern. The rationale is to form a population of place cells as the agent moves around, with the centers placed on the trajectory (Fig 1D). To enforce sparsity in the population vector ${𝐮}^{\text{PC}}$ and clear spatial tuning specificity, a lateral inhibition mechanism compares the cosine similarity between the new weight vector ${𝐖}_{i}^{\text{GC},\text{PC}}$ and the existing non-zero weights ${\text{W}}_{j\neq i}^{\text{GC},\text{PC}}$ against a threshold $\theta_{\text{inh}}^{\text{PC}}$. If it is above threshold, the new place cell is discarded.

The activation of each place cell is calculated using a bounded cosine similarity function, which determines how closely the cell’s tuning curve matches the current grid-cell activity, followed by a generalized sigmoid function. The latter acts as a parametrized filter with β being the gain and α the bias, responsible for the sensitivity and thresholding of the input, affecting the shape of the resulting place field (Fig 1E).

Additionally, for each neuron, an activity trace *m* is recorded with time constant $\tau^{\text{PC}}$, representing the decay of activation over time. A compact summary of the principal model and task parameters is provided in S1 Table. Further details of the implementation, including lateral inhibition and recurrent connectivity, are provided in the Supplementary Information.

### 2.2. Neuromodulation

Neuromodulators deliver event signals: rewards, denoted DA (for dopamine), and boundary collisions, denoted BND. In particular, the latter is an internal variable that correlates with and tracks the sensory experience of mechanical collisions.

They are driven by binary inputs and defined through a leaky variable with exponential decay.

Each modulator *k* updates its connection weights with the place cells through a Hebbian rule based on place cell activity ${𝐮}^{\text{PC}}$ and the error:


$$\begin{array}{c} \Delta {𝐖}^{k}=\eta^{k}{𝐮}^{\text{PC}}\left(v^{k}-{𝐖}^{k}\right) \end{array}$$ (1)

The term in brackets can be interpreted as an error term that implements a simple form of predictive coding, and is inspired by temporal-difference learning [33], aligning with evidence that neuromodulatory systems signal prediction errors and update beliefs [19,55,56]. This mechanism was intended to support robustness to environmental changes, such as reward displacement or the introduction of new obstacles.

The weight vectors are restricted to remain non-negative. Reward modulation tags cells near reward locations, whereas boundary modulation builds a representation of the environmental edges. The result is a set of scalar fields over the place cells, and it forms the core of our model of a cognitive map (Fig 1F). Throughout the manuscript, the architecture is understood as containing only place cells: labels such as reward-modulated (or DA-tagged) and boundary-modulated (or BND-tagged) refer to subsets of place cells carrying non-zero modulatory weights, not to distinct neuron types. See the Supplementary Information for full learning rules and parameter settings.

### 2.3. Modulation of place fields

In addition, we tested whether neuromodulators could directly alter spatial tuning. The place fields were dynamically shifted and resized according to recent salient events, effectively changing the cell density and the area of the receptive fields.

More in detail, following a reward or collision signal, the place field centers were displaced within the grid-cell space according to a vector whose magnitude depended on the neuromodulator $v^{k}$ and the spatial proximity to the reward or collision location:


$$\begin{array}{c} \Delta {𝐖}_{i}^{\text{GC},\text{PC}}=c^{k}v^{k}\varphi_{\sigma^{k}}({𝐮}^{\text{GC}}-{𝐖}_{i}^{\text{GC},\text{PC}}) \end{array}$$ (2)

Here, φ is a Gaussian function with width $\sigma_{b}^{k}$ and $c^{k}$ a scaling factor. This rule is inspired by BTSP plasticity [49], which shifts CA1 place fields following salient experiences. This action was applied only to recently active cells, that is, with an activity trace greater than a given threshold $\theta^{k}$. The motivation was to prioritize activity within a recent time window, consistent with suggestions of dopaminergic gating in the hippocampus [57,58]. Furthermore, this approach is consistent with mechanisms such as the BCM rule [59] and the aforementioned BTSP, both of which rely on asymmetric kernels that reduce or filter out inactive neurons [39].

Lateral inhibition prevents field overlap during remapping. Moreover, field size is modulated by scaling the gain of recently active neurons, allowing neuromodulators to transiently enhance or suppress the spatial sensitivity of specific cells. This modulation rule involves the gain $\beta_{i}$ of each cell being adjusted proportionally to its activity trace $m_{i}$, a reference gain constant $\bar{\beta }$, and a modulatory scaling variable.


$$\begin{array}{c} \beta_{i}=c_{a}^{k}m_{i}\bar{\beta }+(1-m_{i})\bar{\beta } \end{array}$$ (3)

where $c_{a}^{k}$ is a scaling gain parameter, for which a value of 1 means absence of modulation.

Finally, these constitute the mechanisms through which place fields could be reshaped, and the cells that were not affected maintained stable and unchanged fields.

### 2.4. Policy and behavior

To evaluate the navigation ability of the model, we implemented a simple policy that switched between exploration and reward-seeking behavior. The transition between these two modes was based on an external trigger, which indicated whether the reward was ready to be discovered, and the state of the internal map, defined as the presence of non-zero dopaminergic connections. In other words, the purpose of the behavior was dictated by the internal availability of a reward representation; see S2 Fig. Planning was defined as the process of determining a target location, computing a path as a sequence of place cells from the one corresponding to the current position to the one nearest the goal, and executing the actions required to traverse such a sequence. The paths were generated using a graph-based pathfinding algorithm in which place cells served as nodes, synaptic connections as edges, and the calculations involved the operation of population vectors, as shown in Fig 1B. To further improve navigation, we introduced a cost function on the graph nodes, assigning lower values to boundary-modulated cells. This bias discouraged, but did not forbid, paths that approach environmental boundaries, as illustrated in Fig 1G.

Exploration consisted of two possible strategies: a random walk, for purely stochastic movements, and periodic goal-directed navigation toward a randomly selected visited location, aimed at preventing stagnation.

In contrast, exploitation, defined as reward-directed navigation, involved identifying the location of the reward within the cognitive map. This location corresponded to the average position of DA-modulated place cells, reflecting mechanisms such as hippocampal replay and value-based navigation [60–62].

Lastly, wall-avoidance and goal-planning together constitute a way for the model to functionally distinguish and treat reward and collision signals.

## 3. Results

The main objective of this work was to assess the model’s ability to construct an adaptive spatial representation that supports effective goal-directed behavior.

To evaluate the model, we generated a set of closed environments with an increasing number of internal walls, thereby varying the difficulty of mapping and navigation. The internal arrangement of walls and corners renders these environments non-convex. They thus qualify as a wayfinding setting [63], where the goal location is hidden from the agent’s immediate line of sight. At each time step, the agent’s selected action updated its position. A binary collision signal was generated (1 if the new coordinates intersected or crossed a wall, and 0 otherwise). Similarly, the reward signal was defined relative to the goal location, delivering a value of 1 with a probability of $p_{R}=0.8$ when the agent entered a circular region of radius $\sigma_{R}$ around the reward, and 0 otherwise.

A simulation trial was defined by initializing the model without any place cell or learned weights and placing the agent randomly. To facilitate map analysis by increasing the average number of place cells formed, the reward position was initially hidden, becoming available only after a fixed duration. Once available, each time a reward was collected, the agent was randomly teleported to a new location; concurrently, the model’s grid cell modules were re-calibrated to keep the internal map and newly recruited place cells aligned.

We defined the performance metric within a trial as the total number of rewards obtained over a duration *T*_dur_.

This evaluation of map formation and performance within the same trial reflects the online nature of the model, in contrast to approaches based on distinct multi-epoch training and testing stages.

**Fig 2:**
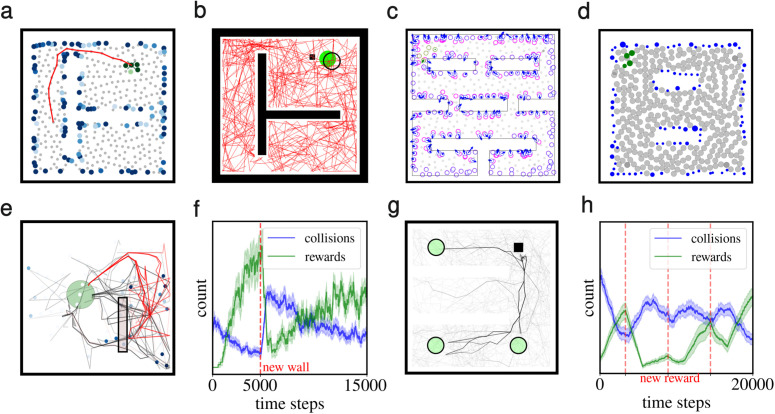
Cognitive maps and performance results. **a**: illustration of a cognitive map composed of boundary-modulated place cells (blue), reward-modulated place cells (green), and a line (red) representing the planned trajectory to reach a target location from the current position. **b**: representation of the same environment as in panel a but with the reward area shown as a circle (green), together with the past trajectories (red line) and the agent position (black square). - **c**: a cognitive map in a different environment showing shifts of the place fields for the two types of modulated place cells. Specifically, the starting boundary-modulated place cell centers (magenta circles), final boundary-modulated place cell centers (blue circles), and their displacement vectors with length proportional to the magnitude (blue arrows) are shown. Similarly, they are shown the starting reward-modulated place cell centers (orange circles), final reward-modulated place cell centers (green), and their displacement vectors (green arrows). Finally, there are also non-modulated place cells (grey filled circles). - **d**: example cognitive map representing place field size modulation. Both reward-modulated place cells (green) and boundary-modulated place cells (blue) are shown as circles whose radius represents the relative width of each cell’s place field, calculated from its gain parameter β: larger β values correspond to smaller fields. This plot is intended as a qualitative visualization; the corresponding statistical comparison across simulations is reported in Fig 3C. - **e**: detour experiment, plot of trajectories before (black) and after (red) the insertion of a wall (rectangle) between the starting and goal positions; the wall is also indicated by the boundary-modulated place cells in blue. - **f**: collision (blue) and reward (green) counts during a simulation trial where a new wall was inserted after 5000 time steps (red dotted line). Curves represent a 1000-step moving average across 64 repetitions, with shaded regions denoting the standard deviation. - **g**: trajectories across multiple trials in an example environment for the reward-relocation task, with the agent starting at the same position (black square) and the reward location periodically moved among the green circles. - **h**: same legend and protocol as in plot f but with the reward location changed instead.

**Fig 3:**
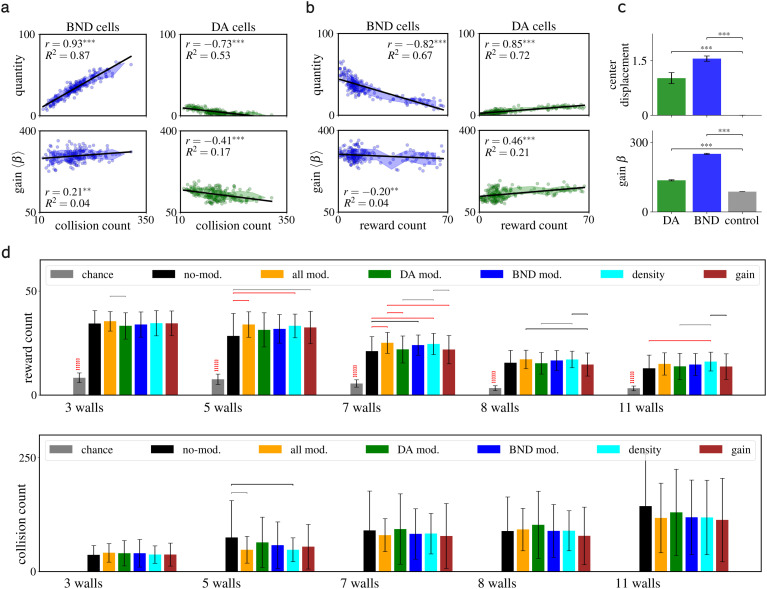
Statistics and performance results. The labels “BND cells” and “DA cells” abbreviate boundary- and reward-modulated place cells, respectively. **a**: effect of collision count on the number of BND- and DA-modulated place cells (top) and on their average activation gain ⟨β⟩(bottom). Each sub-plot reports Pearson’s r, R^2^, and the p-value. - **b**: same layout as in plot a but using reward count. - **c**: Dunnett-corrected differences in place field center displacement (top) and average gain magnitude β (bottom) for DA-modulated, BND-modulated, and non-modulated control place cells; larger β values correspond to narrower place fields. - **d**: reward-count performance across the chance, no-modulation, full-modulation, DA-only, BND-only, density-only, and gain-only variants. Significant pairwise differences are color-coded as: grey <0.05, black <0.01, and red < 0.001. Results obtained from pairwise t-tests across 128 runs with Bonferroni correction; the numerical report is provided in S4 and S5 Tables - **e**: same analysis as plot d but for collision count, calculated from the time a reward representation is initially formed (set at the first non-zero DA weights); the collision count of the control model was not considered, since it lacked reward-driven navigation. The numerical results are reported in S6 and S7 Tables.

Following these simulation settings and parameter optimization, the remainder of this section is organized around three main questions. First, we investigate whether the model can form a cognitive map online to navigate a representative environment (Figs 2A and 2B). Second, we illustrate how this map is locally refined and updated during specific tasks, including place field modulation, detour behavior, and reward relocation (Figs 2C and 2H). Finally, we quantify these event-dependent changes and establish the functional role of neuromodulation via ablation studies (Figs 3A and 3E).

### 3.1. Optimization

The model architecture contains 15 hyperparameters, several of which control non-differentiable components of place cell generation, neuromodulation, and planning. Consequently, we optimized the model using the Covariance Matrix Adaptation Evolution Strategy (CMA-ES) [64] instead of gradient-based methods or manual tuning. The fitness function was designed to simultaneously maximize reward collection and minimize collisions across multiple environments of increasing layout complexity. Full parameter distributions and convergence analyses are detailed in S3 Fig.

### 3.2. Performance in wayfinding

Figs 2A and 2B show a representative simulated trial in which place cells were recruited online during exploration and then used for planned navigation toward the reward. Across the example layouts in Figs 2A and 2D, increasing the number and placement of internal walls could prolong exploration before the cognitive map was sufficiently populated, while also making subsequent route planning more demanding than in simpler layouts.

Fig 2A shows the subsets of place cells tagged by collision (blue) and reward events (green), marking the shape of the boundaries and location of rewards. The composition of these modulated subsets with non-modulated place cells (gray) constitutes what we refer to here as a *cognitive map*. These elements encode the primary spatial and contextual information used during planned navigation. Fig 2B shows the same environment layout (walls in black), the reward region (green), and several representative trajectories (red).

Importantly, the cognitive map was not precomputed but emerged dynamically within a single trial as place cells were sequentially recruited along the exploration trajectory. In stationary environments, previously formed associations between grid and place cells were preserved, restricting map adaptation to newly explored regions. Salient events nevertheless induced local refinement: collisions and reward encounters modulated the activation gain and shifted the place field centers of recently active, tagged cells. Without these modulatory dynamics, the emergent spatial representation remained stable over time, serving as a reliable instrument for navigation.

To overcome the challenges imposed by non-convex layouts, the model utilized graph-based planning over this emergent representation, relying on local spatial information and boundary-modulated place cells, enabling the agent to generate routes around obstacles. The approximately homogeneous distribution of place field centers further allowed graph distances between place cell nodes to approximate the spatial cost of candidate routes.

Overall, this representative trial illustrates how the model can balance reward-directed navigation and obstacle avoidance in a wayfinding setting. Across simulations, early reward localization remained variable because exploration was stochastic, especially in environments with numerous walls and narrow passages.

### 3.3. Local modulation of place field structure

We next investigate how neuromodulation alters local place field properties across different environment geometries. To demonstrate the model’s adaptability across distinct structural constraints, the simulations shown in Figs 2C and 2D utilize different environmental layouts than those in panels A and B. Notably, increasing the number and density of internal walls prolonged the initial exploration phase before the cognitive map was sufficiently populated, while simultaneously rendering subsequent route planning more demanding. One important mechanism driving local adaptation is place field center displacement, which is modulated dynamically by experienced salient events (see Eq. 2). Although the evolutionary optimization did not converge to a unique solution in the parameter space, place cell location modulation regularly produced visible changes in field-center position.

Fig 2C illustrates this field remapping at the conclusion of a trial. Specifically, we highlight the displacement vectors between the initial and final positions of boundary-modulated (magenta to blue) and reward-modulated (orange to green) place cell centers, with arrows indicating the direction and magnitude of the shift. For this representative choice of parameters, boundary-modulated place cells were consistently displaced toward the walls. A possible functional interpretation is that this systematic shift elarges representation space near boundaries, thereby promoting the generation of new place cells to cover open internal areas. Field shifts were also prominent within the reward zone, reflecting the high density of salient encounters in that region during the run.

The second neuromodulatory mechanism involves scaling the neural activation gain (β) via boundary and dopamine-dependent hyperparameters ($c_{a}^{\text{BND}}$, $c_{a}^{\text{DA}}$), directly affecting place field size. Fig 2D provides an illustrative example of this scaling, in which the radius of each circle reflects the relative width of the corresponding place field. Because the gain parameter β regulates the steepness of the activation function, higher gain values correspond to narrower fields. Thus, the compact fields of the modulated cells in Fig 2D visually capture this gain-dependent field shrinkage, a population-level phenomenon quantified below in Fig 3C.

Fig 2E shows the trajectories before (gray) and after (red) wall placement, illustrating both the disruption of the previous paths and the subsequent formation of new place cell tags near the newly added obstacle. Fig 2F complements this example by reporting the average effect of the layout change on reward and collision counts: rewards decreased and collisions increased markedly after wall insertion. These results were averaged over 64 simulation trials, of which 9 failed to reach the reward, yielding a success rate of approximately 85%, a reflection of the unavoidable variance inherent to noisy exploration.

### 3.4. Detour task

We next evaluated the flexibility of the model’s planning mechanisms when subjected to unexpected environmental modifications. To this end, we devised a detour task wherein a novel internal wall was introduced mid-trial. We predicted that relying on a cognitive map containing outdated structural information would initially induce planning failures, thereby forcing the agent to resume stochastic exploration until an alternative route was discovered.

As anticipated, the introduction of the wall caused an immediate, sharp increase in collision counts (Figs 2E and 2F). This elevated sensory error triggered the recruitment of a new subset of boundary-modulated place cells, rapidly updating the internal spatial representation. When the existing cognitive map proved insufficient to resolve a detour, the agent reverted to local exploration until a valid path to the reward zone was discovered. Fig 2E illustrates the trajectories before (gray) and after (red) wall placement, capturing both the disruption of previous paths and the subsequent generation of new place cell tags marking the novel obstacle. Fig 2F complements this spatial trace by visualizing the behavioral impact of the layout change over time; across 64 simulation trials, reward rates dropped considerably while collisions spiked immediately following wall insertion. Of these runs, 9 failed to recover the goal within the trial limit, yielding an overall success rate of approximately 85%, a reflection of the unavoidable variance inherent to noisy, online exploration.

### 3.5. Adaptive goal representation through sensory error

To assess the model’s flexibility to reward displacement as well, we applied a relocation protocol similar to the detour task. Here, the reward location was periodically moved after fixed intervals, requiring the agent to extinguish previous goal associations and discover novel reward zones in a design inspired by computational work [44]. Fig 2G displays representative trajectories as the reward alternated between three distinct locations. The agent consistently transitioned from exploitation and planned navigation at a known site to targeted exploration when a location became unrewarding, as evidenced by the dense overlay of successful navigation paths.

Mechanistically, whenever a planned route resulted in a failed reward prediction, a dopamine-based sensory error signal weakened the synaptic associations between active place cells and the reward representation, driving the rapid extinction of the outdated goal location. Fig 2H tracks the temporal dynamics of this disturbance on performance metrics. Both reward and collision frequencies fluctuated immediately following each relocation event before stabilizing as the agent successfully mapped and exploited the new rewarding zone. These data demonstrate that the architecture effectively recovers from violated sensory expectations.

Several empirical studies support the involvement of dopamine in updating hippocampal spatial representations under shifting reward contingencies. For instance, extinguishing reward expectations reshapes and degrades CA1 spatial maps, an effect replicated by inhibiting ventral tegmental area (VTA) dopaminergic neurons [56]. Likewise, local hippocampal D1/D5 receptor signaling is required for CA1 place cell reorientation following a change in reward-relevant spatial rules [65]. While these findings are broadly consistent with predictive coding and temporal difference learning frameworks [21,33,66], direct empirical evidence for failed reward predictions actively weakening place reward associations precisely as implemented here remains limited. Consequently, we treat our dopamine-based sensory error as a dopamine-inspired, experimentally testable prediction rather than a direct replication of an established hippocampal plasticity rule.

### 3.6. Event-dependent modulation of place cells

After exploring the individual task examples in Fig 2, we quantified the aggregate impact of neuromodulation on place cell populations across multiple simulations. Figs 3A and 3B summarize the results of 50 independent runs across four environments of increasing structural complexity, with each data point corresponding to a single simulation. These plots map the relationships between behavioral metrics, here collision (Fig 3A) and reward counts (Fig 3B), and network features, namely the final count of modulated cells and their average neural activation gains.

The primary trend pattern is a direct relationship between cell count and environmental events: the population of boundary-modulated (BND-tagged) place cells scaled with collision frequency, while dopamine-modulated (DA-tagged) place cells expanded with reward count. Moreover, we also observed inverse relationships for the cross-pairings: high-reward simulations exhibited fewer BND-tagged cells, while high-collision simulations displayed fewer DA-tagged cells.

Overall, these results are not unexpected. Rather than indicating direct cross-modulation dynamics within the network architecture, these inverse relationships are best interpreted as an indirect consequence of the agent’s behavior. High collision counts typically signify prolonged, stochastic exploration, characterized by repeated boundary hits, or difficulty in planned navigation. These more inefficient runs naturally present fewer opportunities to sample the reward zone, leaving the dopamine-tagged sub-population and its associated gain parameters near baseline levels. Conversely, when the reward is discovered early, it rapidly switches from exploratory wandering to efficient and recurrent reward-directed navigation. This transition promotes reward collection while drastically reducing contacts with the walls, thereby limiting both BND-tagged cell recruitment and BND gain updates. The same behavioral coupling accounts for the gain relationships shown in the lower panels of Fig 3B, where the most prominent trend is a positive correlation between reward count and DA gain. This likely reflects a gradual, cumulative gain modulation induced by reward encounters, contrasting with collision-driven BND updates that tend to saturate rapidly due to the high frequency of wall impacts.

We also quantified the local place field alterations illustrated qualitatively in Figs 2C and 2D. Spatial remapping was statistically significant across both modulated cell populations, as evaluated via a corrected Dunnett’s test across 156 simulations (Fig 3C, top). Reward-modulated place cells exhibited an average field-center displacement of 1.02±0.15 (SEM), whereas boundary-modulated cells underwent a larger displacement of 1.56±0.07, both measured relative to a non-modulated control group fixed at zero. Furthermore, boundary-modulated cells received the most pronounced modulatory scaling, reaching an average activation gain of 250.43±2.23 (SEM), compared to 136.15±2.20 for reward-modulated cells and 87.18±0.03 for non-modulated controls (Fig 3C, bottom). The tuning properties of the non-modulated control population remained highly stable, displaying only minor, transient fluctuations proximal to salient sensory boundaries although not enough for them to be BND-tagged.

Functionally, this dual-modulation strategy extends spatial remapping by increasing local neuronal representation density. Mechanistically, sharper place fields reduce the lateral inhibition exerted on neighboring units, allowing additional place cells to be recruited within the same local region. Computationally, this density regulation can be interpreted to opportunely increase the spatial resolution of the cognitive map around critical environmental features. By maximizing representation density near boundaries and narrow passages, the model significantly enhances planning accuracy where navigation margins are tight. These population-level adjustments align with recent empirical findings reporting a higher clustering of place fields near points of behavioral relevance or structural interest, such as environmental boundaries and reward sites, reflecting a biological compromise between spatial resolution and total network size [67–69].

### 3.7. Functional effect of modulation on navigation

We next investigated whether experience-driven modulation directly enhances functional aspects of navigation, specifically maximizing reward collection and minimizing collisions. To isolate the individual contributions of these mechanisms, we conducted a series of ablation experiments where the dopamine (DA) and boundary (BND) modulatory components were systematically disabled, separating their effects on place cell density from their effects on neural activation gain. We evaluated each of the six model variants across five environments of increasing structural complexity, running 128 independent simulations per condition. To put the difficulty of reward collection into perspective, we also evaluated a control variant operating under a random-walk policy.

For all learning architectures, reward and collision frequencies were quantified from the exact moment the agent first discovered the reward to isolate navigation efficiency from initial exploration duration. Statistical significance across configurations was then assessed via pairwise *t*-tests.

The behavioral outcomes illustrated in Figs 3D and 3E show that while all configurations performed above the chance baseline, the magnitude of the performance benefit depended on which modulatory components remained intact. To interpret these results, we focused on specific planned comparisons: the primary test evaluated the full-modulation architecture against the fully non-modulated baseline, while secondary contrasts isolated the independent contributions of reward versus boundary signals (DA-only vs. BND-only) and decoupled the underlying physical mechanisms of adaptation (density-only vs. gain-only).

Our primary comparison revealed that modulation-enabled variants consistently outperformed the control and the fully non-modulated baseline across environments. Despite substantial stochastic variance across individual runs, the non-modulated model harvested significantly fewer rewards.

This global trend suggests that experience-dependent reshaping of the cognitive map provided a behavioral advantage beyond path planning alone.

Among the secondary partial ablations, boundary-related modulation accounted for a noticeably larger share of the navigation benefit than reward-related modulation. This asymmetry suggests that dynamically updating representations of structural walls and narrow corridors is more critical for robust wayfinding than refining representations near known goals, likely because boundary modulation alters the underlying planning graph topology around edges and corners to minimize total path length. Furthermore, the density-modulation variant independently recapitulated most of the performance advantages seen in the full model, whereas gain-only field sharpening yielded weaker and less consistent improvements.

This pattern suggests that the main functional contribution of this neuromodulatory strategy lies not merely in sharpening the tuning curves of an existing population, but in restructuring the spatial density of place cells to better support the planning graph.

Taken together, these ablation studies support the hypothesis that direct, experience-dependent modulation of place field structure can provide a functional advantage for online navigation within complex, non-convex environments.

## 4. Discussion

Exploration and planning in novel and past environments are essential behaviors of animals that directly affect their success in spatial understanding and achieving goals.

An important element behind these abilities is the formation of a map of their surroundings, enriched with information gained from new experiences, known as a cognitive map. In this work, we presented a rate network model that simulates the CA1 area of the hippocampus [10,70]. It differs from previous approaches as it operates online, uses neuromodulated synaptic plasticity, and does not rely on external coordinates.

We used simplified grid cells and synaptic plasticity to generate information-rich spatial representations rapidly forming in novel environments, dynamics directly following experimental observations and computational modeling of place cell generation [71,72]. The architecture and its mechanics are based on the proposal that CA1 place coding is supported by entorhinal grid cell modules that operate at different spatial frequencies [73,74]. In the spirit of minimizing geometric assumptions in the neural space, we treated the generated place network as a topological graph learned through velocity inputs, reminiscent of path integration [14,75,76]. External sensory information was added locally to each graph node, i.e., a place cell, through the action of neuromodulators acting as a signal filter of salient events such as boundary collisions and reward occurrences. We took inspiration from experimental findings about the involvement of dopaminergic projections in hippocampal representation [65,77]. This idea of enriching a spatial map aligns with the concept of a *labeled graph* [78,79], a cognitive map proposal that addresses the problems of geometric and metric consistency by harnessing noisy observations and supporting vector-like operations [80–82].

Although incorporating a richer set of sensory inputs, such as additional self-motion cues, could further improve performance, our goal in this work was to provide a proof of concept. Specifically, our interest was to document the emergence of advantageous computational strategies even from relatively simple modeling assumptions.

The tasks we applied the agent to consisted of an exploratory and exploitative phase, in which the system was tasked to plan and reach reward positions. For simplicity, the first stage relied on a random walk process, as more elaborate policies were outside the scope of this work. This choice had the side effect that the reward was not always discovered, leading to the formation of incomplete maps and impairing performance. However, this constraint did not qualitatively alter our central findings.

The simulation results support the model’s central assumptions, showing the formation of cognitive maps and their encoding of information collected during experience.

Previous work has used path integration with deep neural networks, but required extensive gradient-based training [13,16,17]. Another important difference is that our resulting neural network was composed solely of place cells, although neuromodulated, and no other types of neurons were present. This distinction is justified by the partially different task protocol and internal architecture, which constrained the tuning dynamics and also did not receive visual information as in [15]. Furthermore, our model relied on predefined grid cell layers, which constituted a strong and sufficient inductive bias, and did not have to be learned from scratch. The selection of the goal location was based on dopaminergic connections and a spatial average of the associated neurons. This approach is consistent with experimental observations and dopamine activity recordings in CA1, suggesting its role in stabilizing long-term spatial memories and supporting reward-guided navigation [60,65,83].

An additional relevant aspect is the consideration of the place cell layer as an explicit graph data structure, on which path-planning was applied, meant to be a neural-based variant of the Dijkstra algorithm [84], a popular choice for graph computations. In fact, our algorithm relied on biologically plausible neural operations, such as activity propagation through the network and the use of synaptic activity traces as masks to identify the best neighboring place cells. This structured representation introduced a strong inductive bias, promoting robustness and flexibility across environments with varying layout complexity. Moreover, this lifted the need to learn an approximation of it through network dynamics and rely on a greater variability in neural receptive fields.

A similar approach using boundary-modulated place cells has also been used for applying the successor representation (SR) framework to large environments [85], with the emergence of place and grid cell tuning. More broadly, on the one hand, several aspects of the SR framework align with our model: the distinction of the state map and the reward/boundary signals, and the use of an update rule inspired by temporal difference learning [21–23]. In addition, several studies have investigated biologically plausible implementations [24–26], obtaining close and promising matches with experimental results. On the other hand, the construction principles of our architecture required fewer assumptions about the state space size (the environment area can be almost arbitrarily large), while learning the basis of the state representation (place cells) and the successor matrix (associated with the lateral connectivity) is more direct and immediate than in the standard SR formulation. These modeling choices reflect our interest in the online generation of an explicit place cell map with an associated graph representation, while the SR may also produce non-spatial neural profiles. In fact, the type of cognitive map that emerged in our results was solely composed of neurons with a well-defined tuning curve requiring a single grid cell population vector, thus not demanding multiple spatial observations or learning steps, except for adapting the modulation weights to failed predictions. Finally, our architecture was better suited to integrate and study our formulation of neuromodulated plasticity, given direct access to a spatial map.

Adaptability was tested by occasionally moving the reward position, leading to the generation of an internal prediction error that was used to update its representation on the map. The agent was able to weaken previous associations, return to exploration, and encode new reward locations. This behavioral protocol is similar to previous work [86], in which dopaminergic and cholinergic activity was utilized within a Hebbian plasticity rule to strengthen or weaken reward-associated spatial representations. However, as an alternative to exploiting neuromodulators with opposite valence, we followed the direction of temporal-difference learning and predictive coding, a framework linked to hippocampal representations [19,87] and explored by various computational approaches [88–90]. This preference arose from our focus on using the data encoded in the cognitive map itself, in which each position has an associated neuromodulation value array, encapsulated in the modulatory connection weights. This array functions as an expected sensory experience to compare with the actual sensory experience to obtain a learning signal. This mechanism is aligned with long-standing theories linking neuromodulation, particularly dopamine, with prediction error signaling [31,38,66,91,92]. It also fits within the larger framework of predictive coding, which interprets cognition as the minimization of surprise [90,93], and matches with temporal-difference learning, where the values of internal states are continuously updated through sensory feedback [19,21,33].

However, a limitation of our current implementation is the strong simplification of neuromodulation to externally defined sensory events represented as a binary two-dimensional input.

An important consequence of this choice is the marked limitation of the information about the environment available to the agent, which is effectively restricted to boolean quantities. This operationalization differs substantially from biology, where signals are encoded in a distributed manner and integrated with multiple pathways, providing neuromodulation circuits with high-dimensional neural representations and richer responses [47,57]. Furthermore, neuromodulation in our model only influences value representation and place field modulation, which captures only a subset of the larger roles neuromodulators play in the brain [34].

Lastly, the relevance of active modulation of the neuronal properties of place cells was supported by simulated ablation experiments. The primary finding was the statistically significant role of active boundary modulation, obtained by comparing it with a model variant with disabled neuromodulation. This supports the importance of having a flexible representation of the environment layout.

Further investigation involved testing the effect of density and gain modulation separately. The results showed that altering the density of the place cells affected the total count of the rewards collected. These results are consistent with experimental observations that demonstrate the remapping of place fields following salient events [68,94]. Notably, behavioral time-scale plasticity (BTSP) has been shown to induce shifts in CA1 place fields in response to salient inputs delivered through pathways from the entorhinal cortex [39,49,72,95]. Additional studies further support this view, reporting spatial reorganization of place cells to better emphasize behaviorally relevant information [96,97], as well as changes in firing rate following contextual stimuli [98,99], or other activity processes [100].

The effect was particularly clear for boundary-modulated place cells, which displayed a trend of shifting place cell centers closer to the walls, yielding a statistically significant improvement in performance. One possible interpretation is that a tighter representation of environmental edges leaves more room for the interior, improving its spatial resolution, and thereby enhancing mapping and supporting more efficient path planning. Consistent with this perspective, previous studies have reported an increase in the clustering of place field edges near surface boundaries [101], and have shown that the presence of visual cue edges stabilizes spatial tuning [102]. It should be noted that in our model, the boundary information was limited solely to collision signals rather than feature-rich sensory input. Nevertheless, the emergence of strategies that parallel biological observations suggests the existence of shared computational advantages, supporting previous work documenting the benefit of increased cell clustering near boundaries for spatial accuracy and context discrimination [69,103].

Regarding reward-modulated place cells, despite the reliable experimental evidence to support their remapping, the effect was weak and did not substantially improve behavior, a pattern that likely followed from the baseline constraints of our simulation settings.

Concerning the modulation of place fields, there is significant experimental evidence of their alteration during reward events [104–106], some reporting shrinkage near reward objects [107], and more markedly near boundaries [108]. In this direction, two possible experimental predictions can be made, based on the constraint of using self-motion cues for the generation of cognitive maps.

First, in the model, place field sizes near boundaries are initially approximately homogeneous, and are reshaped only after meaningful events, such as collisions. This can be tested by correlating individual place-field structural metrics with the cumulative count of relevant events occurring near their fields. Secondly, a similar test could be performed for rewarding events, potentially going further by blocking dopamine release in order to assess its causal involvement.

In our setup, the place-field modulation was implemented by scaling the neural activation gain. This led to a general reduction in field size across all modulated cells.

In the case of boundary-modulated place cells, this shrinking effect may account for the improved performance in two ways: first, by increasing the spatial resolution of the environment layout [109]; and second, by freeing up space for other place cells to form nearby. The latter may result from reduced lateral inhibition and the inward shift of the field center toward the boundaries.

However, gain modulation alone did not produce statistically significant effects, suggesting that, in this protocol, it may need to be coupled with density modulation to produce measurable improvements.

For reward place cells, the impact of gain modulation was less pronounced. This may be due to the limits of our experimental protocol or reward signal, with the latter being defined purely in spatial terms and lacking any meaningful non-spatial feature.

Previous studies have proposed that the Locus Coeruleus (LC) is involved in place cell reorganization [41] and overrepresentation near reward locations through the co-release of dopamine and noradrenaline [40]. A possible experimental prediction that emerges from our results is that preventing place cell density modulation may indirectly decrease the quality of reward-directed navigation, as suggested by the remapping of boundary-modulated cells shown in Fig 2C and the statistics reported in Fig 3D.

More specifically, this can be tested in animal models by pharmacological blockade of LC afferent connections to CA1, once a reward has been experienced. Then, performance can be evaluated by considering: the count of rewards fetched; the difference between the animal’s trajectory between the initial and reward location and the theoretical spatial geodesic (shortest path); and the average distance from the walls. However, a relevant confounding variable in this setup is the difficulty of isolating CA1 inputs, alongside the involvement of multiple and complex additional signals in the generation of the cognitive map.

In conclusion, this work showed a possible architecture for coupling emergent spatial representations with neuromodulated plasticity to achieve an experience-driven cognitive map. The reliance on some spatial and algorithmic inductive biases, grid cells, and a planning algorithm supports the idea of a labeled graph for goal navigation. Future work can investigate the application to other spatial domains, such as motor control and three-dimensional navigation. In addition, a richer input feature can be added, such as visual information [110], as well as new neuromodulators that encode different sensory dimensions or internally generated signals.

## Supporting information

S1 Text Supporting information text. (PDF)

S1 Fig Place fields obtained from grid-cell activity. a: grid cell modules with different granularity shown along a continuous trajectory in an open space. For visualization purposes, each module is represented as composed of four sub-modules of 9 grid cells each, whose periodic tuning generates activity that repeats in space. b: place cells whose spatial tuning has been obtained from the concatenation of the grid cells population vector.
(TIF)

S2 Fig Diagram of the behaviour selection process. (TIF)

S3 Fig Distribution of evolved hyper-parameters. - a-o: results from the last generation, from a run with population size of 96 individuals. Each point represents one individual from the final population, with parameter value on the x-axis and average reward count on the y-axis.
(TIF)

S4 Fig Sample of generated environments. (TIF)

S5 Fig Detour-task comparison between the full and modulation-ablated models. (TIF)

S1 Table Summary of principal model and task parameters. (XLSX)

S2 Table Comparison of neural network models for spatial navigation and representation. (XLSX)

S3 Table Post-wall performance in the detour task for the full and modulation-ablated models. (XLSX)

S4 Table Mean reward counts across conditions and environments. Values shown as mean ± SEM.
(XLSX)

S5 Table Significant pairwise comparisons (Bonferroni corrected). (XLSX)

S6 Table Mean collision counts across conditions and environments. Values shown as mean ± SEM.
(XLSX)

S7 Table Significant pairwise comparisons (Bonferroni corrected). (XLSX)
