# Supplementary material for: Flexible navigation with neuromodulated cognitive maps
Source: PLoS Comput Biol. 2026 Jul 28;22(7):e1013487. doi: 10.1371/journal.pcbi.1013487 (PMC13450844; doi:10.1371/journal.pcbi.1013487)
Supplement: Supplementary file 1 [file pcbi.1013487.s001.pdf]

S1 Text

Grid cell module

The model defines a correspondence between the global environment in which the agent moves, a two-dimensional Euclidean space $\mathbf{R}^2$, and a bounded local space of a grid module, corresponding to a torus.

The global velocity $\mathbf{v} = \{x, y\}$ is then mapped to a local velocity, scaled by a speed scalar s_l^{gc} specific to the grid cell module l, which determines its periodicity in space.

The choice of a toroidal space is motivated by well-established experimental evidence of the neural space of grid cells, which are organized in modules of different sizes spanning the animal's environment. However, the shape of their firing pattern is known to be hexagonal, which corresponds to the optimal tiling of a two-dimensional plane, giving rise to a neural space lying on a twisted torus. In this work, for simplicity, we consider a square tiling and thus a square torus, without much loss of generality except for the slight increase of grid cells required for a sufficient cover.

A grid cell module l of size N^{gc} is identified by a set of positions defined over a square centered on the origin with side length 2, such that $\{(x_i, y_i) \mid i \in N^{\text{gc}} \ \wedge \ x_i, y_i \in (-1, 1)\}$. This local square space has boundary conditions for each dimension, such that, for instance, when $x_t + s_l^{\text{gc}} \cdot v_x > 2$ the position update is taken to the other side $x_{t+1} = x_t + s_l^{\text{gc}} \cdot v_x - 2$, where s_l^{gc} is the scale of the velocity in the local space of the module l with respect to the real global agent velocity $v = \{v_x, v_y\}$. When the module is initialized, the starting positions of its cells are uniformly distributed over the square forming a lattice. When the agent is reset in a new position at the beginning of the new trial, a displacement vector is applied to the last cell positions such that the mapping between the module local space and the global environment is preserved.

The firing rate vector of each cell is determined with respect to a 2D Gaussian tuning curve centered at the origin at $(0, 0)$, and it is calculated as

$$r_i = \exp\left(-\frac{x_i^2 + y_i^2}{\sigma_l^{\text{gc}}}\right),$$

where σ_l^{gc} is the width of the tuning curve for module l. An illustration of the receptive field in a 2D environment and a toroidal space is reported in S1 Fig, panels **a** and **b**.

The final population vector of the grid cell network GC is the concatenated and flattened firing rate vector of all modules $\mathbf{u}^{\text{GC}}$.

In our model, each grid cell had a tuning width of 0.04. They were defined as 9 modules of size 36, and the relative speed scales were
$\{1., 0.8, 0.7, 0.5, 0.4, 0.3, 0.2, 0.1, 0.07\}$.

Place cells

Tuning formation The tuning of a new place cell is simply defined as the current GC population vector $\mathbf{u}_t^{\mathrm{GC}}$, and it is assigned the index of the first silent cell, which is added to the forward weight matrix $\mathbf{W}_i^{\mathrm{GC,PC}} \leftarrow \mathbf{u}^{\mathrm{GC}}$.

In order to avoid overlapping of place fields, lateral inhibition is implemented. More specifically, the tuning process is aborted in case the cosine similarity of the new pattern and existing patterns is greater than a threshold $\theta_{\mathrm{inh}}^{\mathrm{PC}}$.

Each cell represents a position in the GC activity space, which can be considered a node within a graph of place cells (PC). Although it is possible to use only the N^{GC}-dimensional tuning patterns and remain agnostic about the dimensionality of the space in which the agent lives, we mapped each pattern to internally derived 2D positions to simplify graph construction, planning, and visualization. Then, the PC recurrent connectivity matrix is calculated with a nearest neighbors algorithm, which instead of a fixed number K of neighbors uses a lateral distance threshold $\theta_{\mathrm{rec}}^{\mathrm{PC}}$.

Activity The current firing rate of the PC population is determined by the cosine similarity between the GC input and the forward weight matrix, then passed through a generalized sigmoid $\phi(z) = [1 + \exp(-\beta(z-\alpha))]^{-1}$. The parameter α represents the activation threshold, or horizontal offset, while β the gain, or steepness.

$$u_i^{\mathrm{PC}} = \phi\left(\cos\left(\mathbf{u}^{\mathrm{GC}}, \mathbf{W}_i^{\mathrm{GC,PC}}\right)\right) \tag{1}$$

An activity trace is also defined, which has an upper value of 1 and decays exponentially:

$$m_i = -m_i/\tau^{\mathrm{PC}} + u_i \tag{2}$$

It is used as a proxy for a memory trace.

In the model, a PC population is defined by its average place field size, determining the granularity of the spatial representation. An example of place-cell layer tuning obtained from a continuous trajectory over a square environment is illustrated in S1 Fig, panel **b**.

Modulation

Neuromodulation is implemented as a reward-sensitive signal, represented as DA (imitating the function of dopamine), and a collision-sensitive signal, represented as BND (for boundary). Its dynamics are defined in terms of a leaky variable v whose state is perturbed by an external input x, whose qualitative meaning differs for each neuromodulator k.

$$\begin{aligned} v^k &= -v^k/\tau^k + x^k \\ v^k &= \max(v^k, 0) \end{aligned} \tag{3}$$

Learning rule The connection weights $\mathbf{W}^k$ are updated according to a plasticity rule composed of a Hebbian term, involving the leaky variable, the place cells that are above a certain threshold θ^k, and the current connection weights value:

$$\Delta \mathbf{W}^k = \eta^k \mathbf{u}^{\mathrm{PC}} \left(v^k - \mathbf{W}^k \right) \tag{4}$$

where η^k is the learning rate: $\eta^{\mathrm{DA}} = 0.9$, $\eta^{\mathrm{BND}} = 0.9$. Additionally, connection values are kept nonnegative.

Active neuronal modulation Neuromodulation acts on the neuronal profile of the place cells by affecting the value of the activation gain and relocating the center of their tuning.

Gain modulation is implemented using activity traces and a constant reference gain value $\bar{\beta}$:

$$\beta_i = c_a^k m_i \bar{\beta} + (1 - m_i)\bar{\beta} \tag{5}$$

where c_a^k is a scaling gain parameter, and if it is 1 then no modulation occurs.

Center relocation is applied to recently active neurons with non-zero trace m_i. For a place cell i with position $\mathbf{W}_i^{\mathrm{GC,PC}}$ (in the grid cell space), a displacement vector q_i is calculated with respect to the current position $\mathbf{u}^{\mathrm{GC}}$:

$$q_i = c_b^k v^k \exp\left(-\frac{\|\mathbf{W}_i^{\mathrm{GC,PC}} - \mathbf{u}^{\mathrm{GC}}\|}{\sigma^k} \right) \tag{6}$$

where c_b^k is a scaling relocation parameter, while σ^k is the width of the Gaussian distance. This displacement is used to move in the GC activity space and get the new GC population vector to use as a tuning pattern.

For this modulatory action, only cells whose activity trace was greater than the modulator-specific threshold θ^k were considered, to prevent the involvement of silent cells. In this case, it is also ensured that the center of the new place field is at a minimum distance $\theta_{\min}^{PC}$ from the others; here, Euclidean distance is used.

Decision making

Behaviour selection logic The possible behaviors are *exploration* and *exploitation*, and an action is defined as a 2D velocity vector. For exploration, an action can be generated either as random navigation, using a polar vector of fixed magnitude (the speed) and angle sampled from a uniform distribution, or as a step within a goal-directed navigation plan to reach a random destination, which corresponds to a randomly sampled existing place cell. During goal-directed navigation, the magnitude of the velocity vector is less than or equal to a fixed speed value, depending on the distance from the next target position in the plan. Instead, for the exploitation behavior, the action is a step within a goal-directed navigation towards the reward location. The behavior selection process depends on the experience of collisions, the presence of a plan,

and the success of navigation planning. A diagram of this logic is shown in S2 Fig.
The positions of the agent and of the target location for planning are identified by the place cells population vector. In particular, the reward position (x_r, y_r) is determined by the weighted average of the centers x_i, y_i of the place cells with respect to their DA-modulated connection weights. Furthermore, only the top five place cells are considered.

$$\begin{aligned} x_r &= \sum_i^5 \mathbf{W}_i^{DA} x_i \\ y_r &= \sum_i^5 \mathbf{W}_i^{DA} y_i \end{aligned} \tag{7}$$

Path-planning algorithm The planning of a new route is implemented as a path-finding algorithm based on the place cell graph, provided as a connectivity matrix C. Its distinguishing feature is the use of a weighting $\widetilde{\mathbf{W}}$ of the nodes according to the neuromodulation map. A description is reported in S1 Algorithm 1.

The principal model and task parameters are reported in S1 Table.

Comparison with previous architectures Several previous computational models show structural and conceptual similarities with the present work. A prominent category among them employs deep neural networks, often with recurrent components, and is based on gradient-based learning strategies such as backpropagation over time. These models typically require multiple training episodes or large datasets for convergence. In contrast, our model adopts biologically inspired local plasticity rules and requires only a single training episode for adaptation.

Other models utilize spiking neurons [1] or explicit neural representations [2], and incorporate online learning rules more closely aligned with ours. These models also focus more directly on goal-directed navigation, in contrast to purely path-integration tasks. However, both of these rely on external spatial coordinates to represent the current position. Our model instead constructs an internal coordinate system by integrating its own velocity output, enabling endogenous spatial tracking.

A summary of this comparison is reported in S2 Table.

Optimization and hyperparameter convergence

The model architecture contains 15 hyperparameters spanning place cell activity, recurrent connectivity, and the two neuromodulatory mechanisms. Because these parameters interact nonlinearly and several of the relevant operations are not differentiable with respect to behavioral performance, we optimized them using the Covariance-Matrix Adaptation Evolution Strategy (CMA-ES) [3]. The

Algorithm 1 S1 Algorithm Activity-based Pathfinding

Require: Connectivity matrix $C \in \mathbb{R}^{n \times n}$, node weights $w \in \mathbb{R}^n$, start node s, end node e
Ensure: A list of nodes forming a (short) path from s to e, or empty if none found

Initialize activity vector $a \leftarrow \mathbf{0} \in \mathbb{R}^n$; set $a_s \leftarrow 1$
Initialize history list $H \leftarrow [\,]$
▷ — *Forward propagation phase* —
for $t = 1$ to `MAX_PATH_DEPTH` **do**
    $a \leftarrow C \cdot a$
    $a \leftarrow a \circ w$ ▷ Element-wise multiplication with node weights
    $a \leftarrow \sigma(4(a - 0.6))$ ▷ Apply sigmoidal activation: $\sigma(x) = \frac{1}{1+e^{-x}}$
    $a_i \leftarrow 0$ if $a_i < 0.1$ (thresholding)
    Append a to H
    if $a_e > 0$ **then**
        break
    end if
end for
if maximum depth reached **then**
    return $[\,]$ ▷ No path found
end if
if $|H| < 3$ **then**
    return $[s, e]$ ▷ Path is trivially short
end if
▷ — *Backward traceback phase* —
Initialize path index stack $G \leftarrow [[e]]$
for $t = 1$ to `MAX_PATH_DEPTH` **do**
    Let $m \leftarrow C_{G[t-1]}$ ▷ Get neighbors of current group
    if $m_s > 0$ **then**
        break
    end if
    $a \leftarrow H[-(t+1)] \circ m$
    Append $\{i : a_i > 0\}$ to G
end for
▷ — *Path reconstruction* —
$P \leftarrow [e]$
for $t = 1$ to $|G| - 1$ **do**
    Initialize $a \leftarrow \mathbf{0} \in \mathbb{R}^n$
    Set $a_i \leftarrow 1$ for all $i \in G[t]$
    $a \leftarrow a \circ C_{P[-1]}$
    if $\sum a = 0$ **then**
        return $[\,]$ ▷ No valid neighbor
    else
        Choose $j \in \{i : a_i > 0\}$ uniformly at random
        Append j to P
    end if
end for
Append s to P
return reverse(P)

objective function jointly maximized reward collection and minimized collision counts after the first reward representation had formed, averaging performance across environments of increasing layout complexity.

S3 Fig reports the parameter distribution in the final CMA-ES population, plotting the value of each parameter against the corresponding agent's average reward count. Most variables show marked convergence, whereas a subset retains broader or quasi-bimodal distributions, suggesting multiple viable strategies within the same performance regime.

The hyperparameters most directly involved in place-cell firing are the activation gain β, activation bias α, and trace time constant τ^{PC}. Together, these parameters shape the sensitivity, offset, and temporal persistence of place-cell activity, thereby influencing place-field width, overlap, and the density of the recruited map. In the optimized populations, β and α converged to relatively narrow ranges, consistent with the emergence of a dense place-cell graph with weak overlap between neighboring fields.

Regarding neuromodulation, the optimized solutions tended to increase gain scaling for both reward-related (c_a^{DA}) and collision-related (c_a^{BND}) events, favoring narrower place fields for recently modulated cells. This tendency is consistent with the field shrinkage effects later quantified in the main text and with experimental observations near behaviorally relevant locations and walls [4, 5]. In contrast, the parameters controlling center relocation exhibited greater diversity, with both attraction toward and repulsion away from the current agent position observed across successful solutions, especially for collision-related modulation. These patterns are broadly compatible with prior observations of reward-related remapping and shifts in hippocampal place-field location [6, 7, 8, 9, 10].

Environments

The game in which the model is tested has been developed with the Pygame Python library, used under the GNU LGPL version 2.1 license and available at `https://github.com/pygame/pygame`. The environment layout consists of a customizable arrangement of vertical and horizontal hard walls with variable length and fixed width. Some samples are shown in S4 Fig.

The reward object is defined as a circle with a size of 5% of the total environment area. When the agent's position is within its boundary, a binary signal $R \sim \mathcal{B}(p_R)$ is drawn from a Bernoulli distribution with probability $p_R = 0.8$. The duration of the reward fetching is set to 2 time steps.

The agent object is defined as a square with a size of 3.3% of the total area of the environment.

The testing protocol was inspired by the behavior of animals that venture into new territories in search of food. It was divided into two parts:

- **exploration phase**: the agent was placed in a random location within the environment for 10,000 time steps. In this phase, the reward is not present. Further, in order to force greater exploration of the environment, every 3000 steps it was teleported to another random location. This ex-

ternal intervention was intended to mitigate randomness in the agent's exploratory behavioral strategy.

- **reward phase**: a reward is inserted at a random location and made available to be discovered. When encountered, the agent is teleported to a random location within the environment and after approximately 100 time steps, it is able to use reward seeking behavior, in the form of goal-directed navigation. The total duration of this phase is set to 20,000 time steps.

An episode is defined as a continuous trajectory during the reward phase, namely a set of time steps starting from when the agent is placed in a position until either it finds the reward or the simulation ends.

After every teleportation event, the agent grid cell modules were calibrated to maintain consistency within the internal representation of the position and the integration of new place cells within the map. The calibration was implemented as a translation of the agent position in the grid space (for each module) from the previous to the new position.

Detour experiment The protocol is modified such that after a fixed number of episodes the layout of the environment is changed, *i.e.* a wall is inserted. This experiment is meant to test the ability to reach the reward location by using the same cognitive map, and possibly update it with the new sensory information, such as the detection of the new boundaries. For simplicity and clearer statistical analysis, the reward probability p_R was set to 1, since fetching uncertainty was outside the scope of the experiment.

Detour-task comparison with modulation ablated In order to provide direct quantitative support for the role of modulation in updating the cognitive map after a change in the environment shape, we repeated the detour experiment with the modulatory mechanisms ablated. However, here we increased the duration of the exploration phase, as well as the periods before and after the environmental change, with the intent of letting performance (reward collection) stabilize for an improved comparison.

Under these conditions, the wall was inserted at two thirds of the simulation, and then following the same protocol as before. S5 Fig shows the time course of performance and the distribution across runs in the post-wall interval, while S3 Table reports a summary of the statistics. The full model (endowed with all modulatory mechanisms) substantially outperformed the ablated model after wall insertion, collecting 217.9 ± 9.0 rewards versus 44.5 ± 6.9 (mean $\pm$ SEM; $n = 64$ per condition; Welch's t-test: $t = 15.29$, $p = 9.5 \times 10^{-30}$, Cohen's $d = 2.70$). These results support the interpretation that local modulation of place cells is functionally important for updating the cognitive map and restoring successful goal-directed navigation after the introduction of a new environmental feature.

Changing reward experiment During the reward phase, the reward location is changed after a fixed duration.

Ablation experiments Here, we report the numerical results, as tables, of the ablation experiments investigating the role of neuromodulation in altering the neural dynamics of place cells.

We carried out pairwise comparisons between all conditions within each environment using independent sample t-tests.

To account for multiple comparisons, we applied Bonferroni correction to the p-values by dividing the statistical threshold alpha ($\alpha = 0.05$) by the number of comparisons performed, thereby controlling the family-wise error rate. For each environment with k conditions, we conducted $k(k-1)/2$ pairwise comparisons. Only comparisons that remained significant after Bonferroni correction (corrected $p < 0.05$) are reported below. Significance levels are shown as * for $p < 0.05$, ** for $p < 0.01$, and *** for $p < 0.001$. Descriptive statistics are presented as mean $\pm$ standard error of the mean (SEM).
Below we report results for the total number of rewards collected, the number of collision events, as well as their relative cross-comparisons.

The descriptive reward-count statistics are reported in S4 Table. The significant reward-count pairwise comparisons after Bonferroni correction are reported in S5 Table. The descriptive collision-count statistics are reported in S6 Table. The significant collision-count pairwise comparisons after Bonferroni correction are reported in S7 Table.

References

[1] Zuzanna Brzosko, Sara Zannone, Wolfram Schultz, Claudia Clopath, and Ole Paulsen. Sequential neuromodulation of Hebbian plasticity offers mechanism for effective reward-based navigation. *eLife*, 6:e27756, 2017.

[2] William de Cothi, Nils Nyberg, Eva-Maria Griesbauer, Carole Ghanamé, Fiona Zisch, Julie M. Lefort, Lydia Fletcher, Coco Newton, Sophie Renaudineau, Daniel Bendor, Roddy Grieves, Éléonore Duvelle, Caswell Barry, and Hugo J. Spiers. Predictive maps in rats and humans for spatial navigation. *Current Biology*, 32(17):3676–3689.e5, September 2022.

[3] Christian Igel, Nikolaus Hansen, and Stefan Roth. Covariance Matrix Adaptation for Multi-objective Optimization. *Evolutionary Computation*, 15(1):1–28, March 2007.

[4] S.N. Burke, A.P. Maurer, S. Nematollahi, A.R. Uprety, J.L. Wallace, and C.A. Barnes. The Influence of Objects on Place Field Expression and Size in Distal Hippocampal CA1. *Hippocampus*, 21(7):783–801, July 2011.

[5] Sander Tanni, William De Cothi, and Caswell Barry. State transitions in the statistically stable place cell population correspond to rate of perceptual change. *Current Biology*, 32(16):3505–3514.e7, August 2022.

[6] Michael I. Anderson and Kathryn J. Jeffery. Heterogeneous Modulation of Place Cell Firing by Changes in Context. *Journal of Neuroscience*, 23(26):8827–8835, October 2003.

[7] Inah Lee, Amy L. Griffin, Eric A. Zilli, Howard Eichenbaum, and Michael E. Hasselmo. Gradual Translocation of Spatial Correlates of Neuronal Firing in the Hippocampus toward Prospective Reward Locations. *Neuron*, 51(5):639–650, September 2006.

[8] Marianne Fyhn, Sturla Molden, Stig Hollup, May-Britt Moser, and Edvard I. Moser. Hippocampal Neurons Responding to First-Time Dislocation of a Target Object. *Neuron*, 35(3):555–566, August 2002.

[9] David Dupret, Joseph O'Neill, Barty Pleydell-Bouverie, and Jozsef Csicsvari. The reorganization and reactivation of hippocampal maps predict spatial memory performance. *Nature Neuroscience*, 13(8):995–1002, August 2010.

[10] Katie C. Bittner, Aaron D. Milstein, Christine Grienberger, Sandro Romani, and Jeffrey C. Magee. Behavioral time scale synaptic plasticity underlies CA1 place fields. *Science*, 357(6355):1033–1036, September 2017.
