# Supplementary material for: Flexible navigation with neuromodulated cognitive maps
Source: PLoS Comput Biol. 2026 Jul 28;22(7):e1013487. doi: 10.1371/journal.pcbi.1013487 (PMC13450844; doi:10.1371/journal.pcbi.1013487)
Supplement: Supplementary file 2 [file pcbi.1013487.s015.pdf]

We thank the reviewers for their insightful and constructive comments. We have performed a major revision of the manuscript, including a restructuring of the Results section for clarity, the addition of comprehensive statistical analyses, and the refinement of our theoretical claims. Detailed responses to specific points follow below in italic.

Reviewer #1 The paper explains a biologically inspired model for the formation and flexibility of cognitive maps, based on path integration, boundaries, and reward locations. The topic is both interesting and important in the field, and the paper makes an interesting attempt to connect place cell dynamics with adaptive navigation behavior. However, several concerns need to be addressed before the paper can be considered for publication. In particular, the results would benefit from further clarification and from more explicit quantitative comparisons.

---

1. One of the stated aims is to show that the model can build spatial maps on the fly, emphasizing online, neuromodulation-driven adaptation. However, this contrast with one-shot learning or offline training is not explicitly shown in the Results. For example, Figure 2a shows a cognitive map. but it is unclear how this map emerges during exploration and how stable it is over time.

We clarified the distinction between 'one-shot' and 'online' learning in the Introduction as well as in the Results. We also updated Figure 1d (formerly 1c) to visually illustrate how place cells are generated sequentially along the agent's trajectory during exploration.

---

2. In the detour task, how does the cognitive map change in response to the insert wall? In the goal changing task, how does the map change in response to the location changes? How do these changes compare to the existing experimental data? Also, it would be good to add explicit performance metric.

When a wall is inserted during a trial, changes to the cognitive map depend on the collision events that occur. Such events involve nearby place cells that were previously not tagged as the boundary (since no wall was detect2ed) and tag them. This new labeling of cells affects the path-finding process, leading to planned trajectories that avoid the new wall. When it is the rewarding area that changes, then it is the reward events that tag new place cells, while the previously-tagged cells lead to prediction errors which in turn determine the weakening of the reward-tags (i.e. DA-place cells weights). These mechanisms are consistent with the associative learning and prediction-error literature (e.g., TD learning; Schultz, 2016). We now explicitly mention this connection in the Results and clarified how reward- and boundary-tagged cells adjust following environmental changes. The performance metric (total reward per trial) is also now stated clearly at the beginning of the Results.

---

3. Re modulation of place field size, it is not clear how figure 2g-h demonstrate that. It would be good to show some stats. Also, where is the red line in figure 2h?

Plot 2g illustrated a cognitive map with place cells having variable field size, additionally, the displacement of the cell centers was reported as red arrow. Plot 2h illustrates the same in a different environment, but without the cells centers. We apologize for the confusion caused by the previous visualization. We have replaced the qualitative plots (former Figs. 2g-h) with a quantitative analysis in Figure 4c. This new figure presents bar plots reporting the statistical differences in place center remapping and gain modulation, using a corrected Dunnett's test.

---

4. The model relies on path integration, but in several experiments the agent is teleported (e.g., after fetching during modulation tests). How does the model maintain map stability under these conditions, given that teleportation disrupts path integration?

The disruption of the internal representation of position was addressed by re-calibrating the position within each grid cell module after each teleportation, preserving the consistency of the grid activity, the place cells activity, and the outer environment. We now clarified this aspect more explicitly at the beginning of the Results section as well as in the Environment subsection in the Supplementary Material.

---

5. Re effect of modulation on performance, what are details for the models? How do you modulate gain, density, etc? How do you define reward- and boundary modulated cells? What are their respective percentages? The text states that there are statistical differences between models, but no stats reported. Phrases, such as 'the statistical difference of most modulation-powered models with respect to…', 'the higher count is noticeable' and 'density modulation… worse than the model…' etc, need to be backed up with quantitative comparisons (e.g., mean ± SEM, statistical tests). Figures 3a–c are not clearly labelled or explained. Similarly, the explanation of Fig. 2d does not match the plot, and Fig. 2e requires clearer description.

We clarified in the Methods how gain and density modulation operate and how reward and boundary-tagged cells are defined. Because their proportions depend on the environment, we now report these relationships in Fig. 3a–b and added quantitative analyses (Figs. 4a–b) plus statistical tables (S1–S5).

---

6. The reward and collision signals are implemented almost the same way, apart from their values. However, reward and boundary signals are quite different: rewards move around, while boundaries are relatively stable. The consequences of this simplification should be discussed. How does the model capture or fail to capture this difference?

We agree that the binary nature of reward and boundary signals represents a strong simplification. In the model, they share similar variable dynamics, they serve distinct functional roles in during planning (goal attraction vs. cost-based avoidance), with consequences on the evolution of their hyperparameters. We now improve the discussion of this limitation and contrast it with biological distributed coding in the Discussion section.

---

7. Re Convergence of evolved hyperparameters, it would be helpful to clarify how each hyperparameters shape place fields, which then further influence the fitness. In particular, how do gain, bias, and trace parameters shape firing patterns, and how do these firing patterns then influence navigation and fitness?

The gain and bias determine the shape of the neural activation function (a generalized sigmoid), while the trace affect the decay of the activation function. We have now mentioned this reference better in the Methods as well as made their relation to the place fields and fitness in the Results.

---

8. The Discussion does a nice job contrasting with SR model. However, it could be made clearer how your model specifically addresses known limitations of SR models, especially in terms of 'one-shot generation of an explicit place cell map'.

We acknowledge that 'one-shot' might be misplaced in this context, since it is usually considered in learning from a few/one example(s) in classification tasks. 'online' is probably better suited, since it directly relates to the training and testing within a single episode/trial. We now added a new sentence re-stating the generation of place cells from a single grid cell activity vector.

---

9. The discussion proposes predictions (e.g., field shrinkage after collisions, LC involvement). It would be useful to explicitly connect them to the figures and results in the paper, so readers can see how the model output motivates those predictions.

We agree with the suggestions, and we inserted a reference to the Figures shown in the Results.

---

10. Overall the results and discussion would be strengthened by a closer link to existing experimental findings.

We acknowledge that the discussion can be improved, we now inserted references to additional studies.

Reviewer #2 The manuscript proposes an interesting framework for online learning of spatial representations. The proposed model is tested in several simulations with the aim of highlighting its capabilities and robustness to change.

I found the manuscript interesting to read but I believe that it requires a major overhaul in terms of clarity and presentation of the ideas.

Major Comments

1. The introduction is rather long and, because of this, in some sections it is slightly unclear which aspects are crucial to the proposed model. The last section of the introduction should be rewritten more succinctly with the aim of briefly describing the model, the aims / hypotheses of your tests and how this was tested.

We have rewritten the final paragraph of the Introduction to be more succinct. We also deeply restructured the Results section with clear subheadings (e.g. 'Detour task', 'Adaptive goal representation') to more directly state the goal of each manipulation before presenting the data.

---

2. Overall, the full manuscript is difficult to follow at times. This is particularly true for the results section. It is often not clear how the figure panels relate to the claims being made, why some results are shown (e.g., Fig. 4), or how they relate to the main aim. I would suggest rewriting the results such that you clearly state the goal of your manipulation/task, what aspect of your model is important for that manipulation, and how the observed results relate to what happens in the brain when such manipulations are used.

We agree with the observation. We followed up by re-organizing the Results section, and improved the readability of its parts.

---

3. There are little to no statistics used to be able to evaluate your claims (e.g., only Fig. 3 uses statistical tests as far as I understand and it is not clear what the results of those tests are). Please make the statistical analysis explicit and clearer.

We agree. We made more explicit the tests used and the reference to the statistical significance. In addition, we deleted and changed some plots in this regard (Figure 3, 4) with hopefully clearer and more informative results.

Minor comments

4. Idiothetic-only input. You motivate the use of idiothetic information with the goal of reducing architectural complexity and proving the validity of the approach with minimal sources of information. We know the EC contains head-direction cells. Please clarify how this choice affects interpretation and whether adding cues would change conclusions.

It is very true that EC and the hippocampus have a great variety of spatial neurons (and many more). In this work, we aimed more to a proof of concept than a closer take on neuroanatomy. We added a sentence in the Discussion to highlight that.

---

5. What motivated the choice of using cosine similarity comparison as a lateral inhibition mechanism? is there modelling work that speaks to this?

Cosine similarity measures the similarity of vectors in terms of an angle and it behaves better when the vector norms can be largely different (which is sometimes the case in our model), unlike using something like the L2 norm (see Gaussian distance). Since it maps to an angle in (-1, +1), coupling it with a sigmoid yields the interval (0, +1), allowing us to ignore when two vector representations are anti-correlated (not useful in our context), and providing a handy and simply parametrized similarity function. There is previous work using it (Chunjieaet al., 2017; Koren er al., 2024; Lagzi et al., 2024)

---

6. While the use of BND as an event signal is interesting, I am not sure whether the reference to serotonin is relevant. Based on the cited literature it is not clear how serotonin would relate to signalling boundary collisions. I suggest rephrasing the citations to be clearer, citing the appropriate literature, or removing these citations and acknowledging that the neural substrates that could implement BND information as defined in the model are not clear.

We agree that the link to serotonin was speculative. We have removed these references from the Methods section to maintain a sufficient adherence to the literature.

---

7. It would be worthwhile to motivate how the choice of modulating place fields based on an activity threshold relates to the literature showing changes in place fields following rewards.

The activity threshold is meant to filter out sufficiently inactive neurons, a practice shared by multiple standard rules in computational modeling, and that can be achieved in several different ways, such as using exponential weight kernel updates. We have now added a few more references in the Methods.

---

8. The authors note that the exploration/reward-seeking behavior was toggled using a policy that depended on an external reward trigger. It seems important to explain this in more detail, considering navigation patterns in the maze determine the learned representations and planning efficiency.

We agree, and now specify better in the Policy paragraph in the Methods.

---

9. Exploration used a random walk. Rodents tend to exhibit spatial biases where they over-prefer to navigate closer to boundaries (walls). It would be useful to acknowledge this as a limitation in the discussion clearly or discuss how it might impact the learned representations.

We have added a statement in the Discussion acknowledging that our use of a random walk for exploration is a simplification compared to the biological bias of rodents to follow boundaries during exploration.

---

10. The description of the model hyperparameter optimization in the naturalistic task section is not very clear. I suggest rewriting it to state what the implications of the chosen strategy are, as someone unfamiliar with this approach (relative to other approaches) will not understand the reasoning.

We agree with the consideration. We improved the description of the evolution search optimization at the beginning of the Results.

---

10. You state your primary aim in the 1st sentence of your results. It would have been easier to follow the manuscript if this were stated succinctly earlier.

We agree, following up on previous comments, we edited the Introduction and Results section in this direction.

---

11. "Whenever a goal path resulted in a failed prediction, the DA-based sensory error weakened the association between the place cells and the reward signal, leading to an extinction of its representation at that location." This is an interesting mechanism; is there empirical evidence that DA would drive similar changes in place field representations? Please clarify.

These principles align on the one hand with predictive coding and temporal difference learning (approaches to decision-making relying on prediction errors), and on the other hand on associative plasticity rules such as Hebbian learning, since a failed prediction can be represented by two previously associated neurons and the subsequent situation in which they fail to be simultaneously active trigger long term depression. From this it can be argued that any synapses with Hebbian-like plasticity behave along these lines. More specifically, to dopamine, midbrain neurons in the VTA are notoriously linked to prediction errors. We have made this connection more clear in the Methods and Discussion.

We agree and made this aim explicit earlier in the Introduction and again in the Results. This framework is aligned with predictive coding, TD learning, and Hebbian plasticity principles. In particular, failed predictions correspond to decorrelated activity and can drive long term depression mechanism. VTA dopamine neurons are well known to encode prediction errors (Lerner et al., 2021). We clarified these connections in the Methods and Discussion.

The authors thank the reviewers for their availability and constructive feedback, which has helped us improve both the clarity and precision of our work. We have done our best to address all the concerns raised in the review and we hope that our revisions meet your expectations. We hope to hear your final decision.

References

Schultz, W. (2016). Dopamine reward prediction error coding. Dialogues in clinical neuroscience, 18(1), 23-32.

Luo, C., Zhan, J., Xue, X., Wang, L., Ren, R., & Yang, Q. (2018, September). Cosine normalization: Using cosine similarity instead of dot product in neural networks. In International conference on artificial neural networks (pp. 382-391). Cham: Springer International Publishing.

Lagzi, F., & Fairhall, A. L. (2024). Emergence of co-tuning in inhibitory neurons as a network phenomenon mediated by randomness, correlations, and homeostatic plasticity. Science Advances, 10(12), eadi4350.

Koren, V., Malerba, S. B., Schwalger, T., & Panzeri, S. (2025). Efficient coding in biophysically realistic excitatory-inhibitory spiking networks. Elife, 13, RP99545.

Lerner, T. N., Holloway, A. L., & Seiler, J. L. (2021). Dopamine, updated: reward prediction error and beyond. Current opinion in neurobiology, 67, 123-130.
