# Supplementary material for: Flexible navigation with neuromodulated cognitive maps
Source: PLoS Comput Biol. 2026 Jul 28;22(7):e1013487. doi: 10.1371/journal.pcbi.1013487 (PMC13450844; doi:10.1371/journal.pcbi.1013487)
Supplement: Supplementary file 3 [file pcbi.1013487.s016.pdf]

Response to Reviewers

We thank the editor and reviewers for their constructive comments and for recognizing the improvements made in the previous revision. We have now revised the manuscript further to clarify the logic of the figures, expand the statistical support, standardize terminology, soften claims that were broader than the evidence presented, and move detailed optimization material to the Supplementary Information.

Reviewer 1

---

1. Regarding the reply to initial comment 1, there is no clear explanation of Figure 3d, the question of how stable the maps are over time has not been addressed, and Figure 3b,c,d,g appear to use different environmental layouts.

We thank the reviewer for pointing out that the revised figure logic and the notion of map stability were still not sufficiently explicit. First, we improve the clarity of Figure 2d (former Figure 3d) in both the caption and the Results text. The revised caption now states that panel d is a qualitative illustration of place field size modulation, that circle radius represents relative place field width, and that larger gain values correspond to narrower fields. We also direct the reader to Figure 3c (former Figure 4c) for the corresponding quantitative comparison, so that Figure 2d is not interpreted as the main statistical evidence. Second, the Results now state more explicitly how the cognitive map emerges online and what we mean by stability in the present model. Particularly, formed grid-place cells associations are usually preserved and unchanged, except for the activation of event-driven modulatory mechanisms that locally may shift and resize place fields. However, globally the map can indeed change in overall extension as new regions are explored. Third, we added an explicit overview of Figure 2 (former Figure 3) explaining why different example layouts are used across panels. Specifically, panels a-b are the same environement, while c-d are chosen both different to showcase how the model reacts to diverse layouts. The complexity of the environments affects the quality and duration of exploration, as well as the ability to plan efficiently.

---

2. Line 335, what does Figure 3r refer to? Regarding the reply to initial comment 2, the descriptions on the map change in response to walls are clear, but modelling data supporting them were not evident.

We thank the reviewer for noting both issues. First, we corrected the erroneous panel reference in the detour subsection. Second, we strengthened the modelling support for wall-induced adaptation in two ways. In the main Results, we now link the detour interpretation more directly to the trajectory and time-course evidence in Figures 2e-f (former 3e-f), clarifying that wall insertion leads to increased collisions, the emergence of a new subset of boundary-modulated place cells, and the eventual recovery of successful navigation once the map is updated. In addition, we added a new direct quantitative comparison between the full model and a modulation-ablated control in the detour task to the Supplementary Information. In this analysis, post-wall reward performance was substantially

higher in the full model than in the ablated model (217.9 ± 9.0 *vs.* 44.5 ± 6.9, *mean* $\pm$ *SEM; Welch's t-test:* $t = 15.29$, $p = 9.5 \times 10^{-30}$, *Cohen's* $d = 2.70$, $n = 64$ *per condition). This data provides direct modelling evidence that local modulation supports map updating and behavioral recovery after environmental change.*

---

3. Regarding initial comment 3, the explanation is clear; however, statistics are still missing when comparing place field sizes, and statistical power is not reported when quantifying the remapping effect.

We agree that the previous version did not make the statistical support sufficiently explicit. In the revised manuscript, we clarified that Figure 3c (former 4c) is the main quantitative analysis for both place field remapping and gain-dependent field-size modulation, whereas Figures 2c-d (former 3c-d) are illustrative examples. We also increased the number of simulations used for these comparisons from 30 to 156 with intent of strenghtening the statistics, and updated both the caption and the Results text accordingly. The revised Results now report the sample size, the use of a corrected Dunnett's test, and the updated mean $\pm$ *SEM values directly in the main text. Specifically, reward-modulated place cells showed an average center displacement of* 1.02 ± 0.15, *boundary-modulated place cells showed an average center displacement of* 1.56 ± 0.07, *and non-modulated place cells served as a zero-displacement control. The corresponding gain values were* 136.15 ± 2.20 *for reward-modulated place cells,* 250.43 ± 2.23 *for boundary-modulated place cells, and* 87.18 ± 0.03 *for non-modulated place cells. Since larger gain values correspond to narrower place fields, these revised analyses provide new quantitative support for the field-size comparison and improve the robustness of the remapping estimates.*

---

4. There are inconsistencies when explaining place cells, reward cells, and boundary cells. The manuscript states that the network is composed solely of place cells, but reward cells and boundary cells are discussed later. It is also unclear whether BND cells and DA cells refer to the same functionally specialized subsets of place cells.

We agree that the previous terminology could be confusing. In the revised manuscript, we now state explicitly in both the Methods and the Discussion that the cognitive maps contain only place cells (and upstream grid cells). Labels such as reward-modulated, DA-tagged, boundary-modulated, and BND-tagged refer to subsets of place cells carrying different modulatory weights, not to distinct neuron classes. We also standardized this terminology throughout the Results and Discussion. Where compact labels such as "BND cells" and "DA cells" remain in figure panels for readability, the corresponding caption now defines them explicitly as abbreviations for boundary- and reward-modulated place cells.

Reviewer 2

---

1. One main recommendation is to give the manuscript to a colleague with a relevant biology or neuroscience background who has not seen it before, to provide feedback on what is and is not clear.

We agree with this recommendation. We have completed this independent read before resubmission with a colleague who has relevant a relevant background. In parallel, the current revision already incorporates a substantial restructuring aimed at improving readability. In particular, the Results now follow the figure order more closely, detailed optimization material has been moved to the Supplementary Information, broad claims have been replaced, and terminology has been better standardized across the manuscript.

---

2. The beginning of the Results section now provides clearer intuition, but the hyperparameter sections interrupt the flow. The reviewer recommends moving this material to the Supplementary Information and/or making it more succinct in the main text.

We agree that the original optimization section interrupted the flow of the Results. In the revised manuscript, we summarized the main-text optimization paragraph to a brief methodological introduction and moved the detailed hyperparameter convergence discussion and associated figure to the Supplementary Information. This restructuring allows the Results section to focus more directly on the biological and behavioral findings, while still preserving the information needed to document model calibration.

---

3. The manuscript stated that the optimized agent demonstrated effective spatial mapping and adaptation, and that performance scaled proportionally with layout complexity. The reviewer asked where these claims were shown and what statistics supported them.

We agree that the original wording was too broad relative to the evidence shown at that point in the Results. In the revised manuscript, we replaced these global claims with narrower, figure-backed statements. The Results section now explicitly links online map formation and wayfinding to Figures 2a-b (former 3a-b), event-dependent and local modulation effects to Figures 3a-c (former 4a-c) and 2c-d (former 3c-d), functional performance effects of modulation to Figures 3d-e (former 4d-e) and the corresponding supplementary statistical tables, and adaptation to environmental or goal changes to Figures 2e-h (former 3e-h). We also removed the stronger claim about performance scaling proportionally with layout complexity, since this effect was not presented as a dedicated statistical analysis and therefore could not be meaningfully justified.

---

4. The reviewer noted that the DA-based sensory-error mechanism is interesting, but

asked for clearer experimental support if DA-based prediction errors are known to weaken associations between place cells and reward signals. If such evidence does not exist, the reviewer suggested framing this as a possible experimental prediction.

We thank the reviewer for this suggestion and agree that the biological status of this mechanism should be stated more carefully. In the revised manuscript, we now cite more direct and relevant evidence that dopaminergic signaling contributes to updating hippocampal reward-related spatial representations, including reward-expectation-dependent reshaping of CA1 maps and the dependence of CA1 place cell reorientation on local dopaminergic signaling. At the same time, we clarify that the exact weakening rule implemented in our model is not presented as an already established CA1 plasticity mechanism with definite neurobiological support. Instead, we now describe the DA-based sensory error as dopamine-inspired, although aligned with known learning paradigms, and as a concrete experimental prediction of the model.

---

5. The labels of figures should coincide with the presentation of the text; for example, the manuscript cited Figure 4c before discussing Figure 4a.

We agree that the previous order of presentation increased the cognitive load on the reader. In the revised Results section, we reordered the subsections so that the text follows the figure logic much more closely. After the initial wayfinding example in Figures 2a-b (former 3a-b), the manuscript now describes the effects of density and field resizing illustrated in Figures 2c-d (former 3c-d), followed by two paragraphs addressing the detour and reward-shift experiments reported in Figures 2e-h (former 3e-h). This change aligns the narrative flow with the figure labels and makes the Results easier to follow.

---

6. The reviewer asked what the negative correlations for the opposite pairings mean, noted that the sign reported in Figure 4 did not match the text, and asked whether these effects persist when reward amount or exploration time is held roughly constant.

We thank the reviewer for noting the inconsistency. We corrected the sign of the reported Pearson correlation coefficients in Figure 4 so that they now match the fitted regression slopes. We also revised the corresponding paragraph to separate the primary, expected event-matched associations from the weaker cross-pairings. The revised interpretation is that the strongest and most direct effects are event matched: collisions recruit boundary-modulated place cells, and rewards recruit reward-modulated place cells. The opposite-sign relationships are now described more cautiously as likely emerging from global exploration and planning dynamics, rather than from a direct mechanistic coupling between the opposite event type and cell recruitment. Specifically, simulations with more collisions tend to have longer exploration phases and less time with stable reward representations for goal navigation; this can coincide with fewer reward-driven place cell modulation. Conversely, simulations in which reward areas are found quickly tend to yield more repeated rewards and less wall hits since goal-navigation is most of the time collision-free, limiting both boundary-modulated cell recruitment (count) and BND-related gain modulation. Because the present revision does not introduce a new controlled analysis in which reward count or exploration time is held constant, we avoid drawing a stronger mechanistic conclusion from these cross-correlations. However we can offer some speculations. On

the one hand, a constant reward amount (in terms of maximum number of rewards obtainable in each trial) would lead the agent to resume exploration once the reward-tagged place cells extinguish their tags (DA weights would go to zero). On the other hand, a constant exploration time if defined as a bounded period with absent reward would simply ensure that some region of the environments are necessarily explored, since no repeated reward-directed navigation can take place. This setup was actually implemented for the statical analysis of the detour experiments, in which a sufficiently extended cognitive map helped factoring out part of the variability inherited by the random exploration.

---

7. The summary of Figures 4d–e covered many panels and bar plots. The reviewer suggested stating the prediction for particular ablations or reducing the number of ablations.

We agree that the original description failed to guide effectively and clearly the reader through the final ablation experiments. We have now dedicated a new paragraph to Figures 3d-e (former 4d-e) and adapted improve their explanations. The revised text now makes clearer the intent to make three main comparisons: full-moulation vs no-modulation; reward-driven modulation (DA) vs collision-driven modulation (BND); density modulation (place centers shift) vs field size modulation (gain scaling). We then summarize only the main takeaways from figure: modulation-enabled models generally outperform the fully non-modulated model, boundary-related modulation contributes more strongly than reward-only modulation, and density modulation captures most of the functional advantages whereas gain-only effects are weaker.

---

8. The reviewer suggested adding a table with the model parameters, brief descriptions, and references from the main text. A similar approach was suggested for the reporting of statistical tests in the Supplementary Information.

We agree that the model relies on many parameters, and that a compact table improves readability. In the revised Supplementary Information, we added a new summary table listing the principal model and task parameters, their role in the architecture, and whether they were evolved or fixed. We also added an explicit pointer to this table in the Methods at the first introduction of the main place cell parameters. In addition, the Results now refer more explicitly to the supplementary statistical tables when discussing the ablation results, so that the connection between the main claims and the underlying tests is easier to follow.

We again thank the reviewers for their careful and constructive feedback. We hope that the revised manuscript and the point-by-point responses above address the remaining concerns.
