# Supplementary material for: Flexible navigation with neuromodulated cognitive maps
Source: PLoS Comput Biol. 2026 Jul 28;22(7):e1013487. doi: 10.1371/journal.pcbi.1013487 (PMC13450844; doi:10.1371/journal.pcbi.1013487)
Supplement: Peer review history

# Peer review: Decision Letter 0

**DOI:** 10.1371/journal.pcbi.1013487.r001

**Authors:** Daniele Marinazzo, Lei Zhang

8 Oct 2025

PCOMPBIOL-D-25-01735

Flexible navigation with neuromodulated cognitive maps

PLOS Computational Biology

Dear Dr. Lepperød,

Thank you for submitting your manuscript to PLOS Computational Biology. Your manuscripit has been seen by two experts in the field. After careful consideration, we feel that it has merit but does not fully meet PLOS Computational Biology's publication criteria as it currently stands. Therefore, we invite you to submit a revised version of the manuscript that addresses the points raised during the review process.

I won't repeat the reviewers' comments as I believe they are self-explanatory. Briefly, I'd like to request you provide more justification, explanation, and clarification of your models and how it can be linked with experimental data. I would also encourage you to make the writing more streamlined.

Please submit your revised manuscript within 60 days Dec 08 2025 11:59PM. If you will need more time than this to complete your revisions, please reply to this message or contact the journal office at ploscompbiol@plos.org. When you're ready to submit your revision, log on to https://www.editorialmanager.com/pcompbiol/ and select the 'Submissions Needing Revision' folder to locate your manuscript file.

Please include the following items when submitting your revised manuscript:

* A rebuttal letter that responds to each point raised by the editor and reviewer(s). You should upload this letter as a separate file labeled 'Response to Reviewers'. This file does not need to include responses to formatting updates and technical items listed in the 'Journal Requirements' section below.

* A marked-up copy of your manuscript that highlights changes made to the original version. You should upload this as a separate file labeled 'Revised Manuscript with Track Changes'.

* An unmarked version of your revised paper without tracked changes. You should upload this as a separate file labeled 'Manuscript'.

If you would like to make changes to your financial disclosure, competing interests statement, or data availability statement, please make these updates within the submission form at the time of resubmission. Guidelines for resubmitting your figure files are available below the reviewer comments at the end of this letter

We look forward to receiving your revised manuscript.

Kind regards,

Lei Zhang

Academic Editor

PLOS Computational Biology

Daniele Marinazzo

Section Editor

PLOS Computational Biology

**Journal Requirements:**

1) Please ensure that the CRediT author contributions listed for every co-author are completed accurately and in full.

At this stage, the following Authors/Authors require contributions: Krubeal Danieli, and Mikkel Elle Lepperød. Please ensure that the full contributions of each author are acknowledged in the "Add/Edit/Remove Authors" section of our submission form.

The list of CRediT author contributions may be found here: https://journals.plos.org/ploscompbiol/s/authorship#loc-author-contributions

2) We ask that a manuscript source file is provided at Revision. Please upload your manuscript file as a .doc, .docx, .rtf or .tex. If you are providing a .tex file, please upload it under the item type u2018LaTeX Source Fileu2019 and leave your .pdf version as the item type u2018Manuscriptu2019.

3) Please provide an Author Summary. This should appear in your manuscript between the Abstract (if applicable) and the Introduction, and should be 150-200 words long. The aim should be to make your findings accessible to a wide audience that includes both scientists and non-scientists. Sample summaries can be found on our website under Submission Guidelines:

https://journals.plos.org/ploscompbiol/s/submission-guidelines#loc-parts-of-a-submission

4) Please upload all main figures as separate Figure files in .tif or .eps format. For more information about how to convert and format your figure files please see our guidelines:

https://journals.plos.org/ploscompbiol/s/figures

5) We notice that your supplementary Figures, Table, and information are included in the manuscript file. Please remove them and upload them with the file type 'Supporting Information'. Please ensure that each Supporting Information file has a legend listed in the manuscript after the references list.

Note: Please cite and label the supplementary table and figures as “S1 Table”, "S1 Figure", S2 Figure" and so forth.

6) Thank you for stating "https://github.com/iKiru-hub/PCNN." Please ensure that your Data Availability Statement is written in full sentence.

Note: If the reviewer comments include a recommendation to cite specific previously published works, please review and evaluate these publications to determine whether they are relevant and should be cited. There is no requirement to cite these works unless the editor has indicated otherwise.

**Reviewers' comments:**

Reviewer's Responses to Questions

Reviewer #1: The paper explains a biologically inspired model for the formation and flexibility of cognitive maps, based on path integration, boundaries, and reward locations. The topic is both interesting and important in the field, and the paper makes an interesting attempt to connect place cell dynamics with adaptive navigation behaviour. However, several concerns need to be addressed before the paper can be considered for publication. In particular, the results would benefit from further clarification and from more explicit quantitative comparisons.

1. One of the stated aims is to show that the model can build spatial maps on the fly, emphasizing online, neuromodulation-driven adaptation. However, this contrast with one-shot learning or offline training is not explicitly shown in the Results. For example, Figure 2a shows a cognitive map. but it is unclear how this map emerges during exploration and how stable it is over time.

2. In the detour task, how does the cognitive map change in response to the insert wall? In the goal changing task, how does the map change in response to the location changes? How do these changes compare to the existing experimental data? Also, it would be good to add explicit performance metric.

3. Re modulation of place field size, it is not clear how figure 2g-h demonstrate that. It would be good to show some stats. Also where is the red line in figure 2h?

4. The model relies on path integration, but in several experiments the agent is teleported (e.g., after fetching during modulation tests). How does the model maintain map stability under these conditions, given that teleportation disrupts path integration?

5. Re effect of modulation on performance, what are details for the models? How do you modulate gain, density etc? How do you define reward and boundary modulated cells? What are their respective percentages? The text states that there are statistical differences between models, but no stats reported. Phrases, such as ‘the statistical difference of most modulation-powered models with respect to…’, ‘the higher count is noticeable’ and ‘density modulation… worse than the model…’ etc, need to be backed up with quantitative comparisons (e.g., mean ± SEM, statistical tests). Figures 3a–c are not clearly labelled or explained. Similarly, the explanation of Fig. 2d does not match the plot, and Fig. 2e requires clearer description.

6. The reward and collision signals are implemented in almost the same way, apart from their values. However, reward and boundary signals are quite different: rewards get moved around, while boundaries are relatively stable. The consequences of this simplification should be discussed. How does the model capture or fail to capture this difference?

7. Re Convergence of evolved hyperparameters, it would be helpful to clarify how each hyperparameters shape place fields, which then further influence the fitness. In particular, how do gain, bias, and trace parameters shape firing patterns, and how do these firing patterns then influence navigation and fitness?

8. The Discussion does a nice job contrasting with SR model. However, it could be made clearer how your model specifically addresses known limitations of SR models, especially in terms of ‘one-shot generation of an explicit place cell map’.

9. The discussion proposes predictions (e.g., field shrinkage after collisions, LC involvement). It would be useful to explicitly connect them to figures and results in the paper, so readers see how the model outputs motivate those predictions.

10. Overall the results and discussion would be strengthened by a closer link to existing experimental findings.

Reviewer #2: The manuscript proposes an interesting framework for online learning of spatial representations. The proposed model is tested in several simulations with the aim of highlighting its capabilities and robustness to change. I found the manuscript interesting to read but I believe that it requires a major overhaul in terms of clarity and presentation of the ideas.

Major Comments

- The introduction is rather long and, because of this, in some sections it is slightly unclear which aspects are crucial to the proposed model. The last section of the introduction should be rewritten more succinctly with the aim of briefly describing the model, the aims / hypotheses of your tests and how this was tested.

- Overall, the full manuscript is difficult to follow at times. This is particularly true for the results section. It is often not clear how the figure panels relate to the claims being made, why some results are shown (e.g., Fig. 4), or how they relate to the main aim. I would suggest rewriting the results such that you clearly state the goal of your manipulation/task, what aspect of your model is important for that manipulation, and how the observed results relate to what happens in the brain when such manipulations are used.

- There are little to no statistics used to be able to evaluate your claims (e.g., only Fig. 3 uses statistical tests as far as I understand and it is not clear what the results of those tests are). Please make the statistical analysis explicit and clearer.

Minor comments

- Idiothetic-only input. You motivate the use of idiothetic information with the goal of reducing architectural complexity and proving the validity of the approach with minimal sources of information. We know the EC contains head-direction cells. Please clarify how this choice affects interpretation and whether adding cues would change conclusions.

- What motivated the choice of using cosine similarity comparison as a lateral inhibition mechanism? is there modelling work that speaks to this?

- While the use of BND as an event signal is interesting, I am not sure the reference to serotonin is relevant. Based on the cited literature it is not clear how serotonin would relate to signalling boundary collisions. I suggest rephrasing the citations to be clearer, citing the appropriate literature, or removing these citations and acknowledging that the neural substrates that could implement BND information as defined in the model are not clear.

- It would be worthwhile to motivate how the choice of modulating place fields based on an activity threshold relates to the literature showing changes in place fields following rewards.

- The authors note that exploration/reward-seeking behavior was toggled via a policy that depended on an external reward trigger. It seems important to explain this in more detail, considering navigation patterns in the maze determine the learned representations and planning efficiency.

- Exploration used a random walk. Rodents tend to exhibit spatial biases where they over-prefer to navigate closer to boundaries (walls). It would be useful to acknowledge this as a limitation in the discussion clearly or discuss how it might impact the learned representations.

- The description of the model hyperparameter optimization in the naturalistic task section is not very clear. I suggest rewriting it to state what the implications of the chosen strategy are, as someone unfamiliar with this approach (relative to other approaches) will not understand the reasoning.

- You state your primary aim in the 1st sentence of your results. It would have been easier to follow the manuscript if this were stated succinctly earlier.

- “Whenever a goal path resulted in a failed prediction, the DA-based sensory error weakened the association between the place cells and the reward signal, leading to an extinction of its representation at that location.” This is an interesting mechanism; is there empirical evidence that DA would drive similar changes in place field representations? Please clarify.

**********

**Have the authors made all data and (if applicable) computational code underlying the findings in their manuscript fully available?**

The PLOS Data policy requires authors to make all data and code underlying the findings described in their manuscript fully available without restriction, with rare exception (please refer to the Data Availability Statement in the manuscript PDF file). The data and code should be provided as part of the manuscript or its supporting information, or deposited to a public repository. For example, in addition to summary statistics, the data points behind means, medians and variance measures should be available. If there are restrictions on publicly sharing data or code —e.g. participant privacy or use of data from a third party—those must be specified.

Reviewer #1: Yes

Reviewer #2: Yes

**********

PLOS authors have the option to publish the peer review history of their article (what does this mean?). If published, this will include your full peer review and any attached files.

If you choose “no”, your identity will remain anonymous but your review may still be made public.

**Do you want your identity to be public for this peer review?**  For information about this choice, including consent withdrawal, please see our Privacy Policy.

Reviewer #1: No

Reviewer #2: No

[NOTE: If reviewer comments were submitted as an attachment file, they will be attached to this email and accessible via the submission site. Please log into your account, locate the manuscript record, and check for the action link "View Attachments". If this link does not appear, there are no attachment files.]

**Figure resubmission:**

While revising your submission, we strongly recommend that you use PLOS’s NAAS tool (https://ngplosjournals.pagemajik.ai/artanalysis) to test your figure files. NAAS can convert your figure files to the TIFF file type and meet basic requirements (such as print size, resolution), or provide you with a report on issues that do not meet our requirements and that NAAS cannot fix.

After uploading your figures to PLOS’s NAAS tool - https://ngplosjournals.pagemajik.ai/artanalysis, NAAS will process the files provided and display the results in the "Uploaded Files" section of the page as the processing is complete. If the uploaded figures meet our requirements (or NAAS is able to fix the files to meet our requirements), the figure will be marked as "fixed" above. If NAAS is unable to fix the files, a red "failed" label will appear above. When NAAS has confirmed that the figure files meet our requirements, please download the file via the download option, and include these NAAS processed figure files when submitting your revised manuscript.

**Reproducibility:**

To enhance the reproducibility of your results, we recommend that authors of applicable studies deposit laboratory protocols in protocols.io, where a protocol can be assigned its own identifier (DOI) such that it can be cited independently in the future. Additionally, PLOS ONE offers an option to publish peer-reviewed clinical study protocols. Read more information on sharing protocols at https://plos.org/protocols?utm_medium=editorial-email&utm_source=authorletters&utm_campaign=protocols

# Author comment: Author response to Decision Letter 1

**DOI:** 10.1371/journal.pcbi.1013487.r002

9 Feb 2026

Attachment Submitted filename: response_reviewers.pdf

# Peer review: Decision Letter 1

**DOI:** 10.1371/journal.pcbi.1013487.r003

**Authors:** Daniele Marinazzo

2 Apr 2026

PCOMPBIOL-D-25-01735R1

Flexible navigation with neuromodulated cognitive maps

PLOS Computational Biology

Dear Dr. Lepperød,

Thank you for submitting your manuscript to PLOS Computational Biology. After careful consideration, we feel that it has merit but does not fully meet PLOS Computational Biology's publication criteria as it currently stands. Therefore, we invite you to submit a revised version of the manuscript that addresses the points raised during the review process.

Please submit your revised manuscript by Jun 02 2026 11:59PM. If you will need more time than this to complete your revisions, please reply to this message or contact the journal office at ploscompbiol@plos.org. When you're ready to submit your revision, log on to https://www.editorialmanager.com/pcompbiol/ and select the 'Submissions Needing Revision' folder to locate your manuscript file.

Please include the following items when submitting your revised manuscript:

* A letter that responds to each point raised by the editor and reviewer(s). You should upload this letter as a separate file labeled 'Response to Reviewers'. This file does not need to include responses to formatting updates and technical items listed in the 'Journal Requirements' section below.

* A marked-up copy of your manuscript that highlights changes made to the original version. You should upload this as a separate file labeled 'Revised Manuscript with Track Changes'.

* An unmarked version of your revised paper without tracked changes. You should upload this as a separate file labeled 'Manuscript'.

If you would like to make changes to your financial disclosure, competing interests statement, or data availability statement, please make these updates within the submission form at the time of resubmission. Guidelines for resubmitting your figure files are available below the reviewer comments at the end of this letter.

We look forward to receiving your revised manuscript.

Kind regards,

Daniele Marinazzo

Section Editor

PLOS Computational Biology

**Journal Requirements:**

Please upload all main figures as separate Figure files in .tif or .eps format. For more information about how to convert and format your figure files please see our guidelines:

https://journals.plos.org/ploscompbiol/s/figures

**Reviewers' comments:**

Reviewer's Responses to Questions

**Comments to the Authors:**

**Please note here if the review is uploaded as an attachment.**

Reviewer #1: The authors have addressed most of my comments. However, there are some required further clarification.

1. Regarding the reply to initial comment 1, there is no clear explanation of figure 3d, and the question of how stable the maps are over time has not been addressed.

Also, it looks to me that Figure 3b,c,d,g have different environmental layouts. Can you explain their differences, such as difficulty levels etc? It is not clear why they are used.

2. Line 335, What does Figure 3r refer to?

Regarding the reply to initial comment 2, the descriptions on the map change in response to walls are clear, but I don’t see any modelling data supporting them.

3. Regarding initial comment 3, the explanation is clear; however, stats are still missing when comparing place field sizes. Also there isn’t stats power when quantifying the remapping effect.

4. In the discussion, there are some inconsistences when explaining place cells, reward cells, and boundary cells. In line 500, it is stated that ‘neural network was composed solely of cells… no other types of neurons were present’. But reward cells and boundary cells are discussed later.

In the results, place cells associated with collision and reward events were mentioned (line 302). BND cells and DA cells were mentioned in line 421. It is unclear whether these labels refer to the same functionally specialised subsets of place cells. If they are intended to describe the same units, the terminology should be standardised throughout the manuscript to avoid confusion

Reviewer #2: I appreciate the authors clarifying the manuscript and taking the suggestions / comments into consideration. I think the manuscript has improved substantially and is much easier to read. However, I also believe there are still issues that need to be fixed.

1. One of my main recommendations would be to give this manuscript to a colleague with a background in biology or neuroscience that is relevant to this work, and who has not seen the manuscript before, to provide feedback on what they find (and do not find) clear. I

2. The beginning of the results section now provides clearer intuition for what is happening to the agent. However, your section on hyperparameters (Optimization, Convergence of the evolved hyperparameters) breaks up the flow of the core ideas you are trying to pursue. I would recommend moving it to supplementary materials and/or writing it much more succinctly to explain why Figure 2. is important for your questions.

3. When you state "The optimized agent demonstrated effective spatial mapping and adaptation, supported by the emergence of a cognitive map that facilitated reliable goal-directed navigation. Notably, the model performed successfully across all tested environments, with performance scaling proportionally to layout complexity"

3a. Where is this part shown "... optimized agent demonstrated effective

spatial mapping and adaptation" and what are the statistics / tests supporting this assertion?

3b. "Notably, the model performed successfully across all tested environments, with performance scaling

proportionally to layout complexity" Similar to above, where is this actually shown? Also, neither of these claims report statistics in the text. I believe that this is true based on some of the tests you report in your later figures, but this is not clear to me as someone who's looked at the manuscript / your figures 2x now.

4. Similar to a comment from my 1st review "Whenever a goal path resulted in a failed prediction, the DA-based sensory error weakened the association between the place cells and the reward signal, leading to an extinction of its representation at that location." I do think this is an interesting mechanism and you do cite more relevant literature on this topic in the introduction.

However, if DA-based sensory errors are known to weaken associations between places cells and reward signals, it would be useful to cite work that supports this idea experimentally. By this I mean work that explicitly shows that DA-based PEs impact place cell representations in HPC. If such work does not exist, it would be useful to highlight this is a possible experimental prediction made by your model.

5. More generally, the labels of figures should coincide with the presentation of the text (e.g. you cite Figure 4c before talking about Figure 4a).

6. "A weaker inverse relationship is also present for the opposite pairing: BND with rewards and DA with collisions. This effect can be explained by considering that in simulations with fewer rewards, more time is spent in exploration, which reasonably increases the chances of collision by venturing in unseen regions, as well as relying on random walks"

What does the negative correlation (note you report that correlation with a positive r in Figure 4) tell us in that case? What happens to these correlations when you hold the reward amount / exploration time roughly constant? Based on the updated manuscript, it is not clear to me if you expected this to happen or whether this is just a consequence of what kinds of cells are generated as a function of environmental movement dynamics.

6. "The results in Figures 4d-eshow that all models performed above chance (in gray). Crucially, most modulation-enabled variants outperformed the fully nonmodulated models..."

This sentence summarizes two panels with 10 subpanels each, where in each subpanel you have 7 bar plots each. That is a lot of information. It would be very worthwhile to present what your prediction was for particular ablations or reduce the total number of ablations.

7. It might be worthwhile to put all your parameters in a table with a brief description of what those parameters are and then any time you mention a parameter in the main text for the first time you can refer the reader to that table. This will primarily help with readability. A similar suggestion could work for the reporting of statistics as I see you have conducted a lot tests in the supplementary materials.

**********

**Have the authors made all data and (if applicable) computational code underlying the findings in their manuscript fully available?**

The PLOS Data policy requires authors to make all data and code underlying the findings described in their manuscript fully available without restriction, with rare exception (please refer to the Data Availability Statement in the manuscript PDF file). The data and code should be provided as part of the manuscript or its supporting information, or deposited to a public repository. For example, in addition to summary statistics, the data points behind means, medians and variance measures should be available. If there are restrictions on publicly sharing data or code —e.g. participant privacy or use of data from a third party—those must be specified.

Reviewer #1: Yes

Reviewer #2: Yes

**********

PLOS authors have the option to publish the peer review history of their article (what does this mean?). If published, this will include your full peer review and any attached files.

If you choose “no”, your identity will remain anonymous but your review may still be made public.

**Do you want your identity to be public for this peer review?**  For information about this choice, including consent withdrawal, please see our Privacy Policy.

Reviewer #1: No

Reviewer #2: No

[NOTE: If reviewer comments were submitted as an attachment file, they will be attached to this email and accessible via the submission site. Please log into your account, locate the manuscript record, and check for the action link "View Attachments". If this link does not appear, there are no attachment files.]

**Figure resubmission:**

-->While revising your submission, we strongly recommend that you use PLOS’s NAAS tool (https://ngplosjournals.pagemajik.ai/artanalysis) to test your figure files. NAAS can convert your figure files to the TIFF file type and meet basic requirements (such as print size, resolution), or provide you with a report on issues that do not meet our requirements and that NAAS cannot fix.

After uploading your figures to PLOS’s NAAS tool - https://ngplosjournals.pagemajik.ai/artanalysis, NAAS will process the files provided and display the results in the "Uploaded Files" section of the page as the processing is complete. If the uploaded figures meet our requirements (or NAAS is able to fix the files to meet our requirements), the figure will be marked as "fixed" above. If NAAS is unable to fix the files, a red "failed" label will appear above. When NAAS has confirmed that the figure files meet our requirements, please download the file via the download option, and include these NAAS processed figure files when submitting your revised manuscript.

**Reproducibility:**

To enhance the reproducibility of your results, we recommend that authors of applicable studies deposit laboratory protocols in protocols.io, where a protocol can be assigned its own identifier (DOI) such that it can be cited independently in the future. Additionally, PLOS ONE offers an option to publish peer-reviewed clinical study protocols. Read more information on sharing protocols at https://plos.org/protocols?utm_medium=editorial-email&utm_source=authorletters&utm_campaign=protocols

# Author comment: Author response to Decision Letter 2

**DOI:** 10.1371/journal.pcbi.1013487.r004

13 Jun 2026

Attachment Submitted filename: response_to_reviewers.pdf

# Editor report: Decision Letter 2

**DOI:** 10.1371/journal.pcbi.1013487.r005

**Authors:** Daniele Marinazzo

15 Jun 2026

Dear Dr. Lepperød,

We are pleased to inform you that your manuscript 'Flexible navigation with neuromodulated cognitive maps' has been provisionally accepted for publication in PLOS Computational Biology.

Before your manuscript can be formally accepted you will need to complete some formatting changes, which you will receive in a follow up email. A member of our team will be in touch with a set of requests.

Please note that your manuscript will not be scheduled for publication until you have made the required changes, so a swift response is appreciated.

IMPORTANT: The editorial review process is now complete. PLOS will only permit corrections to spelling, formatting or significant scientific errors from this point onwards. Requests for major changes, or any which affect the scientific understanding of your work, will cause delays to the publication date of your manuscript.

Should you, your institution's press office or the journal office choose to press release your paper, you will automatically be opted out of early publication. We ask that you notify us now if you or your institution is planning to press release the article. All press must be co-ordinated with PLOS.

Thank you again for supporting Open Access publishing; we are looking forward to publishing your work in PLOS Computational Biology.

Best regards,

Daniele Marinazzo

Section Editor

PLOS Computational Biology

Daniele Marinazzo

Section Editor

PLOS Computational Biology

***********************************************************

# Editor report: Acceptance letter

**DOI:** 10.1371/journal.pcbi.1013487.r006

**Authors:** Daniele Marinazzo

PCOMPBIOL-D-25-01735R2

Flexible navigation with neuromodulated cognitive maps

Dear Dr Lepperød,

I am pleased to inform you that your manuscript has been formally accepted for publication in PLOS Computational Biology. Your manuscript is now with our production department and you will be notified of the publication date in due course.

The corresponding author will soon be receiving a typeset proof for review, to ensure errors have not been introduced during production. Please review the PDF proof of your manuscript carefully, as this is the last chance to correct any errors. Please note that major changes, or those which affect the scientific understanding of the work, will likely cause delays to the publication date of your manuscript.

Soon after your final files are uploaded, unless you have opted out, the early version of your manuscript will be published online. The date of the early version will be your article's publication date. The final article will be published to the same URL, and all versions of the paper will be accessible to readers.

For Research, Software, and Methods articles, you will receive an invoice from PLOS for your publication fee after your manuscript has reached the completed accept phase. If you receive an email requesting payment before acceptance or for any other service, this may be a phishing scheme. Learn how to identify phishing emails and protect your accounts at https://explore.plos.org/phishing.

Thank you again for supporting PLOS Computational Biology and open-access publishing. We are looking forward to publishing your work!

With kind regards,

Anitha Samidurai

PLOS Computational Biology | Carlyle House, Carlyle Road, Cambridge CB4 3DN | United Kingdom ploscompbiol@plos.org | Phone +44 (0) 1223-442824 | ploscompbiol.org | @PLOSCompBiol
